# Exploratory Evaluation of the Statistical Fidelity of Large Language Model-Generated Synthetic Rehabilitation Data for Patients with Hip Fracture

**DOI:** 10.3390/jcm15218700

**Published:** 2026-11-07

**Authors:** Ryo Momosaki, Kenta Ushida, Yoshinori Yamamoto, Akio Shimizu

**Affiliations:** Department of Rehabilitation, Mie University Hospital, 2-174 Edobashi, Tsu 514-8507, Japan

**Keywords:** artificial intelligence, clinical informatics, privacy, statistical fidelity, tabular modeling

## Abstract

**Background/Objectives**: Synthetic data generated from aggregate statistics may support preliminary clinical research when patient-level data access is restricted. However, statistical similarity does not establish clinical validity, analytical utility, or privacy protection. We evaluated three datasets independently generated by large language models (LLMs) for patients with hip fractures. **Methods**: Of 26,637 hospitalization records, 12,130 complete eligible records were identified, and 1000 were reproducibly sampled. Aggregate statistics for 12 variables were provided to three LLMs, each generating one 1000-record dataset. We assessed fidelity to prompted marginal targets, preservation of unsupplied distributional and correlation structures, logical plausibility, and performance in an exploratory regression task predicting discharge Barthel Index scores. **Results**: Mean absolute standardized mean differences (SMDs) were 0.164, 0.027, and 0.001 for GPT, Claude, and Gemini, respectively. Mean absolute differences across 36 correlations were 0.090, 0.113, and 0.105, respectively; maximum correlation discrepancies were 0.411, 0.713, and 0.262. In train-on-synthetic/test-on-real evaluations, R^2^ values were 0.167, −0.080, and 0.146, respectively, compared with 0.262 for five-fold cross-validation on real data. **Conclusions**: The three individual LLM-generated datasets reproduced prompted marginal targets with varying fidelity but did not consistently preserve unsupplied multivariable structures or achieve comparable task-specific performance. These exploratory findings do not establish definitive model rankings, demonstrate clinical validity or privacy protection, or support replacing real patient-level data with synthetic data.

## 1. Introduction

Big data in healthcare provide an essential foundation for clinical epidemiology, healthcare quality assessment, and health policy. However, privacy requirements and data-use agreements may restrict the sharing and secondary use of patient-level data. Synthetic data have gained attention as a means of reproducing the statistical properties of real-world data while addressing these constraints. Potential applications include developing and validating analytical methods, education, information system development, and sharing data structures among researchers. Nevertheless, the suitability of synthetic data as substitutes for real data requires separate evaluation of statistical fidelity, analytical utility, and privacy protection [1,2]. In multicenter research, where access to real-world clinical data is often limited, methods that generate analyzable patient-level data structures without transmitting actual patient records to external services may offer practical value.

Generative models, including generative adversarial networks (GANs), have traditionally been used to generate synthetic healthcare data. More recently, large language models (LLMs) have been investigated as tools for generating tabular health data. Miletic and Sariyar compared LLM-generated tabular health data with data generated by a conditional tabular GAN and reported a tendency toward improved performance as model size increased [3]. LLMs allow variable definitions and generation constraints to be specified in natural language and can generate data without requiring the development and training of a new task-specific generative model.

Researchers have also explored approaches for generating patient-level synthetic data from database descriptions or aggregate information without directly providing real patient records to an LLM. Törnqvist et al. generated synthetic patient data from a description of the target database without using source records and reported that some clinical correlations were preserved [4]. Barr et al. reported that providing GPT-4o with descriptive statistics calculated from real perioperative data substantially improved statistical similarity compared with prompting without these statistics [5].

However, similarity in the means and standard deviations of individual variables does not necessarily indicate that the multivariate structure of patient-level data has been preserved. In clinical data, age, comorbidities, treatment, physical function, and length of stay are interrelated. Although Barr et al. demonstrated univariate statistical similarity, they did not comprehensively examine whether relationships among variables were preserved [5]. Lin et al. reported that preserving the distributions and correlations of real-world data became increasingly difficult as the number of variables increased [6]. Therefore, the extent to which LLMs can reproduce intervariable relationships that were not explicitly provided remains uncertain.

The evaluation of synthetic data should also reflect their intended use. A dataset suitable for teaching data manipulation or testing whether an analysis script executes may be inadequate for estimating clinical associations, developing prediction models, or supporting individual patient decisions. Measures of statistical fidelity describe the resemblance between synthetic and source data but do not establish whether analyses using synthetic data will produce estimates comparable to those obtained from real data. Conversely, demonstrating analytical utility for a specific task does not establish adequate privacy protection. Reviews of synthetic medical data have identified substantial heterogeneity in utility and privacy metrics and noted that privacy is often incompletely evaluated [2]. Comprehensive evaluation frameworks therefore distinguish statistical fidelity, task-specific utility, and privacy risk rather than treating synthetic data quality as a single property [7]. This distinction is particularly important for rehabilitation datasets because variables may have bounded, zero-inflated, skewed, or discrete distributions, and clinically meaningful information is often contained in relationships among functional status, treatment timing, treatment amount, and hospitalization outcomes. These considerations motivated the use of complementary distributional and correlation-based assessments in the present study.

This issue is particularly relevant to rehabilitation, where multiple clinical factors are associated with functional outcomes. Early postoperative rehabilitation is recommended after hip fracture [8]. Studies using Japanese Diagnosis Procedure Combination (DPC) data have reported associations between rehabilitation timing or amount and activities of daily living (ADL) at discharge or time to discharge home [9,10]. Patient characteristics, time to surgery, ADL at admission, rehabilitation timing and amount, ADL at discharge, and length of stay are interrelated during acute treatment and rehabilitation. Therefore, evaluating synthetic data for patients with hip fracture requires assessing not only individual variable distributions but also clinically important relationships involving ADL, rehabilitation dose, and length of stay.

In this study, identical aggregate statistics calculated from a multicenter DPC dataset provided by JMDC Inc. were supplied to GPT-5.6 Sol, Claude Sonnet 4.6, and Gemini 3.8 Flash to generate synthetic clinical data related to rehabilitation in patients with hip fracture. We aimed to evaluate how closely synthetic data generated from aggregate statistics reproduced the statistical properties of real DPC data and to compare fidelity across the three LLMs. We focused particularly on the distributions and interrelationships of ADL, rehabilitation timing and amount, and length of stay. We also considered the findings in relation to practical preparatory steps in rehabilitation research, including developing data-management procedures and analysis code, clarifying variable definitions, and coordinating multicenter workflows before accessing controlled patient-level data. The study did not evaluate direct clinical decision-making, patient prognostication, or treatment-effect estimation.

## 2. Materials and Methods

### 2.1. Study Design and Data Source

We conducted a comparative fidelity study using existing multicenter DPC data as the real-data reference and synthetic datasets generated by three LLMs. The data were obtained from a hospital-based database maintained by JMDC Inc. (Tokyo, Japan), which collects information from multiple Japanese medical institutions. The database contains inpatient and outpatient claims, DPC assessment forms, and selected clinical laboratory results [11]. DPC is a patient classification and per diem payment system used for acute inpatient care in Japan. DPC data include discharge abstracts and administrative claims, with diagnoses recorded using International Classification of Diseases, 10th Revision (ICD-10) codes. Available information includes patient characteristics, diagnoses, surgeries and procedures, admission and discharge details, and clinical measures such as ADL [12]. The JMDC hospital-based database collects DPC Form 1, EF files, H files, and related records, which are linked using anonymized patient identifiers [11]. Candidate patients were those admitted with a hip fracture between 1 January and 29 September 2025.

### 2.2. Study Population and Random Sampling

During the study period, 26,637 hospitalization records for femoral neck fractures (ICD-10 S72.0) or trochanteric fractures (S72.1) were identified. The following exclusion criteria were applied sequentially, and the reported counts were mutually exclusive: 1518 records without eligible surgery (K046 or K081), 764 without inpatient rehabilitation, and 512 involving in-hospital death. After these exclusions, 23,843 records remained (Figure S1). Of these, 11,713 records with missing data for at least one analysis variable were excluded through complete-case analysis, leaving 12,130 eligible records. Missing values were identified for BMI in 6407 records (26.9%), BI at admission in 3557 records (14.9%), and BI at discharge in 4036 records (16.9%); these variable-specific counts overlapped. No missing values were observed for the other nine variables. Because the database extract did not allow reliable identification of repeat admissions, the hospitalization record was used as the unit of analysis. A reproducible sample of 1000 records was selected without replacement using IBM SPSS Statistics version 31. The Mersenne Twister generator was initialized with an index of 12,345, and a uniform random number was assigned to each eligible record. The records were then sorted by the assigned random numbers, and the first 1000 were selected. The sample size of 1000 records was chosen pragmatically for this exploratory study. This sample size allowed each LLM to generate a complete record-level CSV file through its web-based conversational interface while facilitating direct comparisons between the real and synthetic datasets at the same sample size. A formal power calculation was not performed because the study was not designed to test a clinical hypothesis or estimate a treatment effect. To assess the implications of this sampling decision, the characteristics of the selected 1000 records were compared with those of all 12,130 eligible complete records (Table S1).

### 2.3. Variables

The 12 variables were fracture type, surgical procedure, sex, age, Charlson Comorbidity Index (CCI), body mass index (BMI), Barthel Index (BI) at admission, days from admission to rehabilitation initiation, days from admission to surgery, total rehabilitation units during hospitalization, BI at discharge, and length of stay. The CCI quantifies comorbidity burden by assigning weights according to the type and severity of comorbid conditions and summing the weights [13]. In this study, Charlson comorbidity categories were identified using ICD-10 diagnosis codes recorded in the DPC data, and their corresponding weights were summed to calculate the CCI [14].

### 2.4. Information Provided to the LLMs

Using the randomly selected 1000-record real DPC sample, we calculated the mean, standard deviation, minimum, and maximum for continuous variables and the proportion of each category for categorical variables. The continuous-variable statistics provided to the LLMs covered age, CCI, BMI at admission, BI at admission, days to rehabilitation initiation, days to surgery, total rehabilitation units during hospitalization, BI at discharge, and length of stay. Category proportions were provided for fracture type, surgical procedure, and sex. Variable names and coding labels were also supplied. No patient-level DPC data, patient-specific combinations of values, or intervariable correlation coefficients were provided.

### 2.5. Synthetic Data Generation

We used GPT-5.6 Sol (OpenAI), Claude Sonnet 4.6 (Anthropic), and Gemini 3.8 Flash (Google). Synthetic data were generated on 13 September 2026, using the web-based conversational interface of each service. The same prompt, variable definitions, and aggregate statistics were provided to each model, with instructions to generate a tabular dataset of 1000 patients in comma-separated value (CSV) format. The prompt also explicitly instructed each model to consider clinically plausible relationships among variables within individual patients. Correlation coefficients and patient-specific combinations from the real DPC data were not provided. Each model was used for a single generation. The datasets were not regenerated, no follow-up instructions were provided, and the generated values were not modified or postprocessed to improve agreement with the real data. The complete prompt is provided in Text S1. GPT-5.6 Sol was accessed through an OpenAI business plan, Claude Sonnet 4.6 through an Anthropic team plan for scientists, and Gemini 3.8 Flash through a Google corporate account. All three services were configured to opt out of the use of submitted content for model training. Each service returned the complete CSV file directly through its conversational interface. No patient-level real data were uploaded. User-controlled randomization parameters were not available in the interfaces. The authors did not intentionally invoke or instruct the services to use a separate code-execution or data-analysis tool during generation. Because the proprietary interfaces did not disclose all internal processing, the use of any non-user-visible internal computation could not be independently verified.

### 2.6. Synthetic Data Quality Checks

For each generated dataset, we examined the numbers of records and variables, missing data, duplicate rows, integer constraints, permitted BI values (0–100 in 5-point increments), negative timing values, surgery or rehabilitation initiation on or after discharge, and unknown category codes. We also assessed whether the total number of rehabilitation units was compatible with the number of inpatient days available after rehabilitation initiation. Under the Japanese fee schedule, disease-specific rehabilitation is generally reimbursable for up to six units per patient per day and, for designated patients, up to nine units per day [15]. We therefore used nine units per available rehabilitation day as a deliberately permissive upper limit rather than the usual six-unit limit. Available rehabilitation days were conservatively calculated as the length of stay minus the number of days from admission to rehabilitation initiation plus one. A record was flagged if the total number of rehabilitation units exceeded nine multiplied by this value. This rule was intended only to identify internally inconsistent combinations of rehabilitation timing, length of stay, and cumulative units; it was not used to assess treatment appropriateness or classify a record as clinically impossible. We also reported combinations of trochanteric fracture and hemiarthroplasty as atypical patterns requiring review rather than as invalid records. Generated records were neither removed nor corrected. Detailed results of these checks are provided in Table S4.

### 2.7. Fidelity of Categorical Variables

For fracture type, surgical procedure, and sex, we compared the category proportions in each synthetic dataset with those in the real DPC dataset.

### 2.8. Statistical Fidelity of Continuous Variables

For continuous variables, statistical fidelity between the real DPC data and each synthetic dataset was assessed using the absolute standardized mean difference (SMD) and the standardized 1-Wasserstein distance. The SMD standardizes the difference in means by the variability in the two datasets; its absolute value was used to assess the magnitude of the discrepancy regardless of direction. Values closer to zero indicate greater agreement in means. The 1-Wasserstein distance quantifies the separation between two entire distributions and reflects differences in distributional location and shape. Because this distance depends on the measurement unit and scale, each variable was standardized using the mean and standard deviation of the real DPC data before the distance was calculated. This measure is referred to as the standardized Wasserstein distance, with values closer to zero indicating smaller distributional discrepancies. Both measures were calculated for each of the nine continuous variables, and their means across these variables were used to summarize the overall discrepancy for each model. Variable-level results with bootstrap intervals are provided in Table S2.

### 2.9. Fidelity of the Intervariable Correlation Structure

To assess the fidelity of intervariable correlations that were not explicitly provided to the LLMs, Spearman rank correlation coefficients (ρ) were calculated for the real and synthetic datasets across all 36 pairwise combinations of the nine continuous variables (9 × 8/2). For each pair, we calculated the signed difference between the synthetic- and real-data correlation coefficients (synthetic minus real), which is reported in Table S3 to show the direction of the discrepancy. The absolute value of this difference was used to assess its magnitude regardless of direction, with values closer to zero indicating greater fidelity. For each model, the mean absolute difference across all 36 pairs was calculated as a summary measure of overall correlation fidelity. Results for all 36 pairs are provided in Table S3.

### 2.10. Statistical Analysis

Random sampling and descriptive analyses were performed using IBM SPSS Statistics version 31. Comparative analyses were conducted using Python 3.13.5, pandas 2.2.3, and NumPy 2.3.5. Nonparametric 95% percentile bootstrap intervals were calculated using 1000 resamples with a random seed of 20261010 for each SMD, standardized Wasserstein distance, Spearman correlation coefficient, correlation difference, and standardized regression coefficient. These intervals quantify resampling uncertainty within the fixed datasets rather than variability across LLM-generated datasets. An exploratory task-specific analysis was conducted using discharge BI as the outcome and the other 11 variables as predictors in an ordinary least-squares model. We compared standardized coefficients, five-fold cross-validated performance within each dataset, and performance when trained on synthetic data and tested on real data, using root mean square error (RMSE) and R-squared. Because the absolute SMD and Wasserstein distance are nonnegative and may be affected by finite-sample bootstrap bias near zero, a percentile interval is not mathematically required to contain the point estimate. Accordingly, these intervals were interpreted as measures of resampling uncertainty rather than uncertainty in model generation. The 11 predictors were age, sex, fracture type, surgical procedure, Charlson Comorbidity Index, body mass index, Barthel Index at admission, days from admission to rehabilitation initiation, days from admission to surgery, total rehabilitation units during hospitalization, and length of stay. This regression analysis was not prespecified in the original study plan and was treated as a post hoc exploratory analysis. For each model trained on a synthetic dataset, the predictor and outcome means and standard deviations estimated from that synthetic training dataset were used for standardization. The same synthetic-data predictor means and standard deviations were applied to the real-data predictors, and predictions were transformed back to the original Barthel Index scale using the outcome mean and standard deviation from the corresponding synthetic training dataset.

### 2.11. Ethical Considerations

The DPC database provided by JMDC Inc. was fully anonymized, and the investigators could not identify individual patients. Before the study, the Ethics Committee of Mie University Hospital reviewed the study and formally determined that ethical review was not required for this secondary analysis of fully anonymized data. No reference or approval number was assigned to this determination. Submitting features or aggregate statistics derived from JMDC data to external commercial LLM services is not permitted on a blanket basis and requires case-specific confirmation and approval. Accordingly, the authors consulted JMDC regarding this study and obtained approval to submit derived aggregate statistics, without patient-level records, to the commercial LLM services. This approval did not authorize third-party distribution of the generated synthetic datasets.

## 3. Results

The real DPC dataset and all three synthetic datasets comprised 1000 patients and 12 variables. No missing values, BI values outside the valid range of 0–100 points, or negative day values were identified in any synthetic dataset. No records in the GPT-5.6 Sol or Gemini 3.8 Flash datasets had days to rehabilitation initiation equal to or greater than the length of stay, whereas one such record was identified in the Claude Sonnet 4.6 dataset. Descriptive statistics are presented in Table 1. All BI values in the generated datasets were integers and multiples of five; the upper quartile of 26.25 for admission BI in the Gemini dataset resulted from quartile interpolation between permitted integer values. The conservative rehabilitation-intensity screening criterion flagged 24 records in the Claude dataset (2.4%) and none in the real, GPT, or Gemini datasets. One Claude record had rehabilitation initiation on or after discharge. Combinations of trochanteric fracture and hemiarthroplasty occurred in 44 GPT records (4.4%), four Claude records (0.4%), and none in the real or Gemini datasets; these combinations were considered atypical rather than impossible (Table S4).

For categorical variables, the proportions of fracture type, surgical procedure, and sex generated by Gemini 3.8 Flash matched those in the real DPC data. The corresponding proportions generated by GPT-5.6 Sol and Claude Sonnet 4.6 were also close to those in the real data, with maximum absolute differences of 1.5% and 1.0%, respectively.

### 3.1. Statistical Fidelity of Continuous Variables

Gemini 3.8 Flash showed minimal discrepancies from the means and SDs provided in the prompt. However, some variables differed from the real DPC data in their medians and IQRs. For example, BI at admission in the real DPC data was 15.9 ± 26.6 points, with a median of 5 [IQR, 0–15], whereas the Gemini dataset had a mean of 15.9 ± 26.6 points and a median of 0 [IQR, 0–26.25]. GPT-5.6 Sol showed comparatively larger differences from the real DPC data in the descriptive statistics for BI at admission, total rehabilitation units, and length of stay. Additional distributional diagnostics are provided in Table S6.

Across the nine continuous variables, the mean absolute SMDs were 0.164 for GPT-5.6 Sol, 0.027 for Claude Sonnet 4.6, and 0.001 for Gemini 3.8 Flash. Thus, GPT-5.6 Sol had the largest mean discrepancy, whereas Gemini 3.8 Flash had the smallest. The corresponding mean standardized Wasserstein distances were 0.253, 0.093, and 0.088, respectively (Table 2 and Table S2). For GPT-5.6 Sol, the largest absolute SMD was observed for BI at admission (0.536), whereas the largest standardized Wasserstein distance was observed for length of stay (0.441). For Claude Sonnet 4.6, the corresponding variables were CCI (absolute SMD, 0.065) and days to rehabilitation initiation (standardized Wasserstein distance, 0.117). For Gemini 3.8 Flash, the largest absolute SMD was observed for days to rehabilitation initiation (0.002), whereas the largest standardized Wasserstein distance was observed for BI at admission (0.234).

### 3.2. Fidelity of the Intervariable Correlation Structure

Correlations for 10 selected variable pairs are presented in Table 3, and results for all 36 pairs are provided in Table S3. Complete correlation and correlation-difference heatmaps are provided in Figures S2–S8. Across all 36 Spearman correlations, the mean absolute difference from the real DPC data was 0.090 for GPT-5.6 Sol, 0.113 for Claude Sonnet 4.6, and 0.105 for Gemini 3.8 Flash. The largest mean absolute difference was observed for Claude Sonnet 4.6, whereas the smallest was observed for GPT-5.6 Sol. Among the 36 pairs, GPT-5.6 Sol produced correlations closest to the real-data values for 13 pairs, Claude Sonnet 4.6 for 12 pairs, and Gemini 3.8 Flash for 11 pairs. The maximum absolute discrepancies were 0.411 for GPT-5.6 Sol, 0.713 for Claude Sonnet 4.6, and 0.262 for Gemini 3.8 Flash. Sign reversals occurred in 12, 15, and 15 of the 36 pairs, respectively. The 10 pairs shown in Table 3 were selected for concise presentation because they represented clinically relevant relationships involving age, BMI, functional status, treatment timing, rehabilitation amount, and length of stay; the complete results are provided in Table S3.

For selected variable pairs, the correlation between total rehabilitation units and length of stay was ρ = 0.729 in the real DPC data, compared with 0.407 for GPT-5.6 Sol, 0.016 for Claude Sonnet 4.6, and 0.837 for Gemini 3.8 Flash. The correlation between BI at admission and BI at discharge was ρ = 0.357 in the real data, compared with 0.257, 0.600, and 0.581, respectively. Similarly, the correlation between days to rehabilitation initiation and days to surgery was ρ = 0.295 in the real data, compared with 0.705, 0.775, and 0.422, respectively.

### 3.3. Representativeness and Exploratory Task-Specific Utility

All 1000 sampled records matched records in the complete cohort of 12,130 records. The mean absolute SMD across continuous variables and categorical levels was 0.042. Most characteristics showed absolute SMDs below 0.10; however, BMI showed an absolute SMD of 0.137, indicating some imbalance between the selected sample and the complete eligible cohort. In five-fold cross-validation, the discharge-BI model trained on real data had an RMSE of 28.84 and an R-squared of 0.262. When models trained on the GPT, Claude, and Gemini synthetic datasets were evaluated on the real sample, the respective RMSE values were 30.64, 34.89, and 31.03, and the corresponding R-squared values were 0.167, −0.080, and 0.146. The standardized coefficient for admission BI was 0.285 in the real-data model and 0.647 in the Claude model, whereas the coefficient for total rehabilitation units was 0.007 and 0.487, respectively (Table S5A). Although Claude achieved an internal five-fold cross-validated R-squared of 0.634, its R-squared when trained on synthetic data and tested on real data was −0.080 (Table S5B). These findings indicate that strong internal performance on synthetic data did not necessarily translate into comparable performance on real data.

In 1000 simulated simple random samples of 1000 records drawn without replacement from the complete cohort, the 2.5th, 50th, and 97.5th percentiles of the maximum absolute SMD were 0.030, 0.056, and 0.092, respectively. None of the simulated samples had a maximum absolute SMD equal to or greater than the observed value of 0.137 (empirical *p* < 0.001).

## 4. Discussion

In this study, three LLMs generated rehabilitation-related synthetic clinical data for 1000 patients with hip fractures using only aggregate statistics from multicenter DPC data, and their statistical fidelity was compared with that of the real DPC data. The principal findings were that all three models generated structured synthetic datasets without receiving real patient-level data; fidelity in reproducing univariate means and distributions varied among models; and correlations that were not explicitly supplied were reproduced to some extent. However, fidelity differed across models and variable pairs.

The ability to generate synthetic data resembling real data from aggregate statistics is consistent with the perioperative study by Barr et al. [5], which reported that adding descriptive statistics to the GPT-4o prompt substantially improved univariate statistical similarity. Nevertheless, despite receiving identical aggregate statistics, the three models in our study differed in fidelity, with GPT-5.6 Sol showing comparatively large discrepancies in BI at admission, total rehabilitation units, and length of stay. Differences in the target dataset, variable structure, prompt design, and model generation process may have contributed to these differences; however, our study design cannot identify the responsible factors. Thus, providing aggregate statistics does not ensure equivalent statistical fidelity across LLMs.

Gemini 3.8 Flash reproduced the supplied means and standard deviations with exceptionally close agreement and had the smallest mean standardized Wasserstein distance. However, for BI at admission, near-perfect agreement in the mean and SD coexisted with discrepancies in the median and IQR. Matching explicitly provided means and SDs therefore does not ensure that the entire distribution of real patient data is reproduced. Synthetic data should be assessed separately for fidelity, analytical utility, and privacy [2,7], as quality cannot be established using a single metric. The proprietary conversational interface did not reveal whether the near-exact agreement with several prompted targets involved internal computation that was not visible to the user; therefore, the underlying mechanism cannot be inferred from the exported CSV alone.

Although most sample-to-cohort differences were small, the observed BMI imbalance exceeded the 97.5th percentile of the maximum absolute SMD in the empirical random-sampling comparison. This comparison was restricted to the 12,130 complete records. Because BMI was missing in 6407 otherwise eligible records and complete-case selection was used, it does not establish representativeness relative to all clinically eligible records, and complete-case selection bias remains possible.

Previous evidence regarding the preservation of intervariable relationships is inconsistent. Törnqvist et al. reported that some clinical correlations were maintained without providing the original data to the LLM [4]. However, Lin et al. found that preserving distributions and correlations became more difficult as dimensionality increased [6]. In another study, Barr et al. compared neurosurgical synthetic data generated by GPT-4o and a GAN and demonstrated that bivariate relationships and clinical analysis results were broadly preserved, although some effect sizes varied [16]. In our study, GPT-5.6 Sol had the smallest mean absolute difference across all 36 correlations; however, the model that produced the closest value for each pair varied among GPT-5.6 Sol, Claude Sonnet 4.6, and Gemini 3.8 Flash. Thus, correlation fidelity depends on both the model and the variable pair, and a single summary measure should be interpreted cautiously. Sign reversals also require contextual interpretation because many occurred in variable pairs with real-data correlations close to zero; a change in sign alone does not demonstrate a reversal of a clinically important association.

Although no correlation coefficients were supplied, all three models generated some correlations in the same direction as those observed in the real DPC data. This finding may reflect medical knowledge acquired during pretraining and the instruction to generate clinically plausible patient-level relationships. However, the effects of pretrained knowledge and prompt instructions were not experimentally separated; therefore, the underlying mechanism remains speculative.

From a rehabilitation perspective, model-dependent differences in clinically important relationships, particularly those between total rehabilitation units and length of stay and between BI at admission and BI at discharge, are relevant. Early rehabilitation is recommended after hip fracture [8], and Japanese DPC studies have demonstrated associations between rehabilitation timing and amount and ADL at discharge and time to home discharge [9,10]. Accordingly, synthetic rehabilitation data should be assessed not only for similarity in marginal distributions but also for the preservation of clinically important relationships involving ADL, rehabilitation dose, and length of stay. For example, the correlation between days to rehabilitation initiation and days to surgery was 0.295 in the real data but 0.705, 0.775, and 0.422 in the GPT-5.6 Sol, Claude Sonnet 4.6, and Gemini 3.8 Flash datasets, respectively. This distortion in a clinically relevant timing relationship illustrates that close agreement with prompted marginal statistics does not ensure preservation of unsupplied intervariable relationships.

Generating patient-level synthetic data from aggregate statistics without transmitting real patient records to an external LLM may have practical value for clinical research involving rehabilitation real-world data. Multicenter clinical data may be subject to data-use agreements and privacy protections that restrict external sharing, making it challenging to reproduce analytical environments across research teams. Synthetic data generated from aggregate statistics could facilitate analytical code development, preliminary testing of analytical procedures, education, communication of data structures, and preparation of analytical workflows before collaborative research begins. This approach may be particularly useful in rehabilitation research, which frequently involves interrelated measures of ADL, rehabilitation timing and amount, and length of stay. However, the present study assessed only one exploratory, task-specific regression analysis and did not establish general agreement in analytical results or practical effectiveness for these applications. We also did not evaluate reidentification risk, and labeling a dataset as synthetic does not guarantee privacy protection. If intervariable relationships differ from those in the real data, association analyses or predictive models fitted to synthetic data may produce different estimates. Therefore, the present findings do not support using these data for clinical decision-making, patient prognostication, or the immediate replacement of real patient data. Fidelity, utility, and privacy should be assessed separately for each intended use [2,7].

### 4.1. Clinical Implications for Rehabilitation Research

The principal clinical implication of these findings is operational rather than therapeutic. In multicenter rehabilitation studies, a schema-matched synthetic dataset could be used before secure access to real data to implement cohort definitions, construct derived variables, test analysis code, prepare tables and figures, and resolve differences in variable interpretation between clinicians and analysts. This preparatory use may be particularly valuable when governance or contractual requirements restrict the sharing of patient-level records across institutions. By helping research teams identify technical and definitional problems at an earlier stage, synthetic data may facilitate the initiation of clinically focused studies while ensuring that substantive estimates and conclusions are derived from appropriately governed real data.

These potential applications are limited to research preparation. They require structural plausibility and sufficient statistical similarity for the intended task but do not necessarily require synthetic data to reproduce every clinical effect estimate. In contrast, using synthetic data to select prognostic factors, estimate treatment effects, compare facilities, or train models intended for patient care would require substantially stronger evidence. For these applications, investigators would need to demonstrate that prespecified analyses produce conclusions consistent with those obtained from held-out real data, including agreement in effect direction, magnitude, uncertainty, calibration, and subgroup performance. The discrepancies in correlation structures observed in this study illustrate why similarity in marginal summaries cannot serve as a substitute for such validation. Accordingly, synthetic datasets should clearly document their generation methods, evaluation results, intended uses, and prohibited uses. This use-specific approach links technical evaluation to practical clinical research applications while avoiding the implication that statistical similarity alone establishes clinical validity.

### 4.2. Priorities for Future Evaluation

Future studies should evaluate repeated generations from each model to distinguish variability due to stochastic generation from differences related to model architecture or prompt design. Prespecified replications across different sample sizes, numbers and types of variables, disease populations, and healthcare databases would help clarify the robustness and generalizability of the findings. Analytical utility should be evaluated using tasks that reflect realistic rehabilitation research, such as estimating associations between rehabilitation timing or dose and functional outcomes, reproducing multivariable regression coefficients, and assessing predictive performance using train-on-synthetic/test-on-real designs. These analyses should be prespecified before model-specific results are examined to reduce the risk of selectively emphasizing favorable outcomes. Privacy should also be explicitly assessed through appropriate disclosure-risk, membership-inference, and attribute-inference evaluations. In addition, external clinical review may identify implausible combinations that distributional metrics fail to detect. A staged evaluation framework could begin with data integrity and range checks, proceed to univariate and multivariate fidelity, assess task-specific analytical utility, and conclude with privacy and clinical plausibility assessments by experts. Only datasets that meet purpose-specific thresholds should be considered for the corresponding applications. This approach would improve clinical interpretability and help determine whether aggregate-statistic prompting can support the reproducible and responsible sharing of rehabilitation data structures across institutions.

Evaluation protocols should also prespecify how missingness, structural zeros, bounded scales, integer-valued variables, and logical constraints involving dates or treatment quantities will be handled. Reporting the numbers of invalid or clinically inconsistent records alongside summary fidelity measures would help distinguish statistically similar datasets from those that are operationally usable. When feasible, prompts, generated data, and analysis code should be archived to facilitate transparent comparisons across model updates and independent replications.

### 4.3. Limitations

This study has several limitations. First, each LLM was used for only one generation; therefore, within-model variability was not assessed. The results should be interpreted as outputs generated on 13 September 2026, under the specific prompt conditions used, rather than as a general ranking of model performance. Second, the study was limited to 1000 patients with hip fractures and 12 variables, and the generalizability of the findings to other diseases, data structures, higher-dimensional datasets, or different sample sizes remains unknown. Third, only one exploratory regression analysis predicting discharge BI was evaluated. Thus, the findings do not establish general analytical utility for other association analyses, predictive models, treatment-effect estimates, or subgroup analyses. Fourth, privacy risks, including reidentification, membership inference, and attribute inference, were not examined. Fifth, commercial LLMs are continuously updated; consequently, using a service with the same name in the future may not reproduce the same output. Sixth, 11,713 of the 23,843 otherwise eligible records (49.1%) were excluded because of missing BMI or BI data, and complete-case selection bias cannot be ruled out. Therefore, the representativeness comparison applies to the 12,130 complete records rather than to all clinically eligible records. Seventh, repeat admissions could not be identified, meaning that the records did not necessarily represent unique individuals. Eighth, the pragmatic sample size of 1000 records may have affected the precision of the fidelity estimates; the findings do not establish whether similar results would be obtained with other sample sizes. The nine-unit-per-day rule was a conservative administrative consistency check based on the available variables and should not be interpreted as a comprehensive clinical validation of rehabilitation delivery. Ninth, no CTGAN, copula-based generator, or independent-marginal statistical baseline was included. CTGAN and conventional copula approaches generally require patient-level training data or a supplied dependence structure, unlike the aggregate-only input used in this study. Conversely, constructing an independent-marginal baseline using only means, standard deviations, minima, and maxima would require arbitrary distributional assumptions for bounded, discrete, skewed, and zero-inflated variables. Consequently, this study does not establish that LLMs offer any advantage over conventional synthetic-data methods. Tenth, five-fold cross-validation within the real dataset and train-on-synthetic/test-on-real evaluation address different performance questions and should not be interpreted as a fully matched head-to-head comparison. In the train-on-synthetic/test-on-real analysis, the real evaluation dataset was the same 1000-record sample from which the aggregate statistics supplied to the LLMs had been calculated. Although no patient-level records were provided to the LLMs, the evaluation data were therefore not fully independent of the information used for synthetic-data generation. Finally, although the analysis code is provided for methodological transparency, the reported analyses cannot be independently rerun from the submitted reproducibility package alone because neither the real patient-level data nor the generated synthetic datasets can be distributed under the applicable data-use conditions. Authorized input datasets are required separately.

## 5. Conclusions

In this exploratory evaluation, the outputs generated by three LLMs using aggregate statistics reproduced the prompted marginal targets to varying degrees but showed substantial discrepancies in distributional, correlation, and regression structures that were not explicitly supplied. These findings apply only to the specific prompts, datasets, access date, and model states examined and do not support a general ranking of model performance. Synthetic data generated from aggregate statistics may be useful for limited preparatory activities, such as analytical code development and education; however, the evaluated datasets should not be used for effect estimation, prediction, subgroup inference, or patient care. Statistical fidelity, task-specific utility, clinical plausibility, and disclosure risk must be independently evaluated for each intended use.

## Figures and Tables

**Table 1:** Descriptive statistics for the real DPC data and each LLM-generated synthetic dataset.

Variable	Real	GPT-5.6 Sol	Claude Sonnet 4.6	Gemini 3.8 Flash
Age, years	85.6 ± 7.6; 87 (81–91)	85.8 ± 7.3; 86 (81–91)	85.6 ± 7.5; 86 (80–91)	85.6 ± 7.6; 87 (81–91)
CCI	0.9 ± 1.1; 1 (0–1)	0.9 ± 1.4; 0 (0–1)	0.9 ± 1.0; 1 (0–1)	0.9 ± 1.1; 1 (0–1)
BMI at admission, kg/m^2^	20.3 ± 2.9; 20.1 (18.2–22.2)	20.5 ± 2.7; 20.5 (18.7–22.3)	20.3 ± 3.0; 20.2 (18.3–22.3)	20.3 ± 2.9; 20.4 (18.4–22.3)
BI at admission	15.9 ± 26.6; 5 (0–15)	4.9 ± 11.7; 0 (0–0)	14.4 ± 25.1; 0 (0–20)	15.9 ± 26.6; 0 (0–26.25)
Days from admission to rehabilitation initiation	2.6 ± 2.4; 2 (1–3)	2.6 ± 3.1; 2 (1–4)	2.5 ± 2.2; 2 (1–3)	2.6 ± 2.4; 2 (1–3)
Days from admission to surgery	2.9 ± 3.3; 2 (1–4)	2.5 ± 2.5; 2 (1–3)	3.0 ± 3.2; 2 (1–4)	2.9 ± 3.3; 2 (1–4)
Total rehabilitation units during hospitalization	35.6 ± 44.0; 22 (13–40)	47.7 ± 44.8; 34 (18–62)	36.1 ± 44.7; 22.5 (12–43)	35.6 ± 44.0; 21 (11.8–42)
BI at discharge	46.5 ± 33.6; 45 (15–75)	46.4 ± 25.2; 45 (30–65)	46.9 ± 34.5; 40 (15–80)	46.5 ± 33.6; 45 (15–75)
Length of stay, days	31.8 ± 22.4; 24 (18–36)	39.7 ± 16.9; 36 (28–48)	31.3 ± 21.0; 26 (17–40)	31.8 ± 22.4; 26 (16–42)
Fracture type: Femoral neck fracture	521 (52.1%)	510 (51.0%)	528 (52.8%)	521 (52.1%)
Fracture type: Trochanteric fracture	479 (47.9%)	490 (49.0%)	472 (47.2%)	479 (47.9%)
Surgical procedure: Open reduction and internal fixation	603 (60.3%)	618 (61.8%)	593 (59.3%)	603 (60.3%)
Surgical procedure: Hemiarthroplasty	397 (39.7%)	382 (38.2%)	407 (40.7%)	397 (39.7%)
Sex: Female	784 (78.4%)	772 (77.2%)	778 (77.8%)	784 (78.4%)
Sex: Male	216 (21.6%)	228 (22.8%)	222 (22.2%)	216 (21.6%)

Continuous variables are presented as mean ± standard deviation (SD) and median [interquartile range (IQR)], and categorical variables are presented as *n* (%). Abbreviations: BMI, body mass index; CCI, Charlson Comorbidity Index; DPC, Diagnosis Procedure Combination; LLM, large language model; BI, Barthel Index.

**Table 2:** Statistical fidelity of continuous variables between the real DPC data and each LLM-generated synthetic dataset.

Variable	Model	Absolute SMD	Standardized Wasserstein Distance
Age, years	GPT-5.6 Sol	0.016	0.112
Age, years	Claude Sonnet 4.6	0.010	0.113
Age, years	Gemini 3.8 Flash	0.001	0.075
CCI	GPT-5.6 Sol	0.039	0.236
CCI	Claude Sonnet 4.6	0.065	0.063
CCI	Gemini 3.8 Flash	0.000	0.047
BMI at admission, kg/m^2^	GPT-5.6 Sol	0.049	0.111
BMI at admission, kg/m^2^	Claude Sonnet 4.6	0.009	0.085
BMI at admission, kg/m^2^	Gemini 3.8 Flash	0.001	0.105
BI at admission	GPT-5.6 Sol	0.536	0.414
BI at admission	Claude Sonnet 4.6	0.057	0.110
BI at admission	Gemini 3.8 Flash	0.000	0.234
Days from admission to rehabilitation initiation	GPT-5.6 Sol	0.013	0.215
Days from admission to rehabilitation initiation	Claude Sonnet 4.6	0.028	0.117
Days from admission to rehabilitation initiation	Gemini 3.8 Flash	0.002	0.027
Days from admission to surgery	GPT-5.6 Sol	0.148	0.172
Days from admission to surgery	Claude Sonnet 4.6	0.026	0.099
Days from admission to surgery	Gemini 3.8 Flash	0.001	0.056
Total rehabilitation units during hospitalization	GPT-5.6 Sol	0.271	0.321
Total rehabilitation units during hospitalization	Claude Sonnet 4.6	0.011	0.048
Total rehabilitation units during hospitalization	Gemini 3.8 Flash	0.000	0.054
BI at discharge	GPT-5.6 Sol	0.002	0.255
BI at discharge	Claude Sonnet 4.6	0.013	0.097
BI at discharge	Gemini 3.8 Flash	0.000	0.058
Length of stay, days	GPT-5.6 Sol	0.398	0.441
Length of stay, days	Claude Sonnet 4.6	0.027	0.101
Length of stay, days	Gemini 3.8 Flash	0.000	0.135
Mean across nine variables	GPT-5.6 Sol	0.164	0.253
Mean across nine variables	Claude Sonnet 4.6	0.027	0.093
Mean across nine variables	Gemini 3.8 Flash	0.001	0.088

Values closer to zero for both the absolute SMD and standardized Wasserstein distance indicate smaller discrepancies from the real DPC data. Abbreviations: SMD, standardized mean difference; CCI, Charlson Comorbidity Index; BI, Barthel Index.

**Table 3:** Fidelity of intervariable correlation structures between the real DPC data and each LLM-generated synthetic dataset.

Pair	Real	GPT-5.6 Sol	Claude Sonnet 4.6	Gemini 3.8 Flash
Age, years—BI at admission	−0.186	−0.173	0.005	−0.190
Age, years—BI at discharge	−0.383	−0.221	−0.104	−0.248
BMI at admission, kg/m^2^—BI at discharge	0.071	0.053	0.021	0.100
BI at admission—BI at discharge	0.357	0.257	0.600	0.581
Days from admission to rehabilitation initiation—Days from admission to surgery	0.295	0.705	0.775	0.422
Days from admission to rehabilitation initiation—Length of stay, days	0.114	0.161	0.018	0.253
Days from admission to surgery—Length of stay, days	0.249	0.169	0.013	0.215
Total rehabilitation units during hospitalization—BI at discharge	0.179	0.279	0.443	0.094
Total rehabilitation units during hospitalization—Length of stay, days	0.729	0.407	0.016	0.837
BI at discharge—Length of stay, days	0.163	0.077	−0.006	−0.089

Note: ρ denotes Spearman’s rank correlation coefficient. Abbreviations: BI, Barthel Index.

## Data Availability

Real-world patient-level data are not publicly available because they were obtained from JMDC Inc. under a data-use agreement. Access requires a separate application, review, and contract with JMDC Inc. The complete generation prompt, supplementary analysis tables and figures, analysis code, and a README describing the reproducibility package are provided with the revised submission. The generated synthetic CSV files are not provided because the JMDC data-use conditions restrict third-party distribution of JMDC data and outputs derived from those data, and JMDC did not authorize distribution of the generated datasets. No real patient-level DPC data are included in the package.

## Supplementary Materials

The following supporting information can be downloaded at: https://www.mdpi.com/article/10.3390/jcm15218700/s1. Text S1: Complete generation prompt and supplied aggregate statistics; Table S1: Full cohort and sample comparisons; Table S2: Fidelity metrics with bootstrap confidence intervals; Table S3: Pairwise Spearman correlations; Table S4: Operational plausibility checks with nonzero findings; Table S5A: Exploratory discharge BI standardized coefficients; Table S5B: Exploratory discharge BI predictive performance; Table S6: Distributional diagnostics; Figure S1: Patient-selection flow; Figure S2: Real DPC data correlation heatmap; Figure S3: GPT correlation heatmap; Figure S4: Claude correlation heatmap; Figure S5: Gemini correlation heatmap; Figure S6: GPT minus real-data correlation-difference heatmap; Figure S7: Claude minus real-data correlation-difference heatmap; Figure S8: Gemini minus real-data correlation-difference heatmap; analysis code.

## References

[1] Gonzales A., Guruswamy G., Smith S.R. (2023). Synthetic data in health care: A narrative review. PLoS Digit. Health.

[2] Kaabachi B., Despraz J., Meurers T., Otte K., Halilovic M., Kulynych B., Prasser F., Raisaro J.L. (2025). A scoping review of privacy and utility metrics in medical synthetic data. npj Digit. Med..

[3] Miletic M., Sariyar M. (2024). Large language models for synthetic tabular health data: A benchmark study. Stud. Health Technol. Inform..

[4] Törnqvist M., Zucker J.-D., Fauvel T., Lambert N., Berthelot M., Movschin A. (2025). A text-to-tabular approach to generate synthetic patient data using LLMs. Proceedings of the 2025 IEEE 13th International Conference on Healthcare Informatics (ICHI).

[5] Barr A.A., Quan J., Guo E., Sezgin E. (2025). Large language models generating synthetic clinical datasets: A feasibility and comparative analysis with real-world perioperative data. Front. Artif. Intell..

[6] Lin Y., Yu Z., Lee S.A. (2025). A case study exploring the current landscape of synthetic medical record generation with commercial LLMs. Proc. Mach. Learn. Res..

[7] Hernandez M., Osorio-Marulanda P.A., Catalina M., Loinaz L., Epelde G., Aginako N. (2025). Comprehensive evaluation framework for synthetic tabular data in health: Fidelity, utility and privacy analysis of generative models with and without privacy guarantees. Front. Digit. Health.

[8] Min K., Beom J., Kim B.R., Lee S.Y., Lee G.J., Lee J.H., Lee S.Y., Won S.J., Ahn S., Bang H.J. (2021). Clinical practice guideline for postoperative rehabilitation in older patients with hip fractures. Ann. Rehabil. Med..

[9] Uda K., Matsui H., Fushimi K., Yasunaga H. (2019). Intensive in-hospital rehabilitation after hip fracture surgery and activities of daily living in patients with dementia: Retrospective analysis of a nationwide inpatient database. Arch. Phys. Med. Rehabil..

[10] Kato D., Tarasawa K., Abe K., Fushimi K., Fujimori K. (2026). Impact of rehabilitation on time to home discharge after hip fracture surgery: A retrospective observational study using the Japanese nationwide database of Diagnosis Procedure Combination. Phys. Ther. Res..

[11] Nagai K., Tanaka T., Kodaira N., Kimura S., Takahashi Y., Nakayama T. (2020). Data resource profile: JMDC claims databases sourced from medical institutions. J. Gen. Fam. Med..

[12] Yasunaga H. (2024). Updated information on the diagnosis procedure combination data. Ann. Clin. Epidemiol..

[13] Charlson M.E., Pompei P., Ales K.L., MacKenzie C.R. (1987). A new method of classifying prognostic comorbidity in longitudinal studies: Development and validation. J. Chronic Dis..

[14] Quan H., Sundararajan V., Halfon P., Fong A., Burnand B., Luthi J.-C., Saunders L.D., Beck C.A., Feasby T.E., Ghali W.A. (2005). Coding algorithms for defining comorbidities in ICD-9-CM and ICD-10 administrative data. Med. Care.

[15] Ministry of Health, Labour and Welfare of Japan Medical Fee Schedule: General Provisions for Disease-Specific Rehabilitation Fees. Patients May Receive up to Six Units per Day, or up to Nine Units per Day when Designated Criteria Are Met. https://www.mhlw.go.jp/web/t_doc?dataId=84aa9729&dataType=0&pageNo=12.

[16] Barr A.A., Guo E., Karmur B.S., Sezgin E. (2025). Synthetic neurosurgical data generation with generative adversarial networks and large language models: An investigation on fidelity, utility, and privacy. Neurosurg. Focus.

