# Supplementary material for: Exploratory Evaluation of the Statistical Fidelity of Large Language Model-Generated Synthetic Rehabilitation Data for Patients with Hip Fracture
Source: J Clin Med. 2026 Nov 7;15(21):8700. doi: 10.3390/jcm15218700 (PMC13764035; doi:10.3390/jcm15218700)

Table S1. Full cohort and sample comparisons

Variable	Level	Full cohort	Sample	Absolute SMD
Age		85.34	85.63	0.039
CCI		0.91	0.93	0.015
BMI		20.74	20.33	0.137
Admission_BI		15.47	15.88	0.016
Days_to_Rehabilitation		2.68	2.60	0.034
Days_to_Surgery		3.00	2.93	0.023
Rehabilitation_Units		34.29	35.61	0.030
Discharge_BI		47.47	46.46	0.030
Length_of_Stay		30.97	31.84	0.040
Fracture_Type	S72.0	54.39	52.10	0.046
Fracture_Type	S72.1	45.61	47.90	0.046
Surgical_Procedure	K046	56.32	60.30	0.081
Surgical_Procedure	K081	43.68	39.70	0.081
Sex	Female	78.66	78.40	0.006
Sex	Male	21.34	21.60	0.006

Continuous entries are means; categorical entries are percentages. Full cohort n = 12,130; sample n = 1,000.

Model labels used throughout the supplementary material and figures are GPT = GPT-5.6 Sol (OpenAI), Claude = Claude Sonnet 4.6 (Anthropic), and Gemini = Gemini 3.8 Flash (Google).

Table S2. Fidelity metrics with bootstrap confidence intervals

Model	Variable	Absolute SMD (95% CI)	Standardized Wasserstein (95% CI)
GPT	Age	0.016 (0.002–0.107)	0.112 (0.087–0.157)
GPT	CCI	0.039 (0.002–0.129)	0.236 (0.179–0.308)
GPT	BMI	0.049 (0.002–0.140)	0.111 (0.071–0.184)
GPT	Admission_BI	0.536 (0.463–0.599)	0.414 (0.364–0.456)
GPT	Days_to_Rehabilitation	0.013 (0.002–0.100)	0.215 (0.139–0.345)
GPT	Days_to_Surgery	0.148 (0.067–0.234)	0.172 (0.127–0.238)
GPT	Rehabilitation_Units	0.271 (0.181–0.364)	0.321 (0.239–0.431)
GPT	Discharge_BI	0.002 (0.002–0.099)	0.255 (0.220–0.294)
GPT	Length_of_Stay	0.398 (0.311–0.503)	0.441 (0.390–0.509)
Claude	Age	0.010 (0.001–0.104)	0.113 (0.079–0.170)
Claude	CCI	0.065 (0.005–0.149)	0.063 (0.029–0.139)
Claude	BMI	0.009 (0.001–0.102)	0.085 (0.070–0.150)
Claude	Admission_BI	0.057 (0.005–0.140)	0.110 (0.094–0.154)
Claude	Days_to_Rehabilitation	0.028 (0.002–0.109)	0.117 (0.091–0.181)
Claude	Days_to_Surgery	0.026 (0.001–0.110)	0.099 (0.068–0.169)
Claude	Rehabilitation_Units	0.011 (0.002–0.102)	0.048 (0.045–0.141)
Claude	Discharge_BI	0.013 (0.001–0.102)	0.097 (0.082–0.147)
Claude	Length_of_Stay	0.027 (0.002–0.117)	0.101 (0.076–0.148)
Gemini	Age	0.001 (0.002–0.100)	0.075 (0.056–0.137)
Gemini	CCI	0.000 (0.002–0.098)	0.047 (0.035–0.116)
Gemini	BMI	0.001 (0.001–0.094)	0.105 (0.077–0.172)
Gemini	Admission_BI	0.000 (0.002–0.102)	0.234 (0.189–0.297)
Gemini	Days_to_Rehabilitation	0.002 (0.002–0.094)	0.027 (0.033–0.121)
Gemini	Days_to_Surgery	0.001 (0.001–0.099)	0.056 (0.048–0.138)
Gemini	Rehabilitation_Units	0.000 (0.001–0.096)	0.054 (0.050–0.131)
Gemini	Discharge_BI	0.000 (0.001–0.102)	0.058 (0.053–0.121)
Gemini	Length_of_Stay	0.000 (0.002–0.103)	0.135 (0.109–0.184)

Table S3. Pairwise Spearman correlations

Model	Variable 1	Variable 2	Real rho (95% CI)	Synthetic rho (95% CI)	Signed difference (95% CI)	Sign reversal
GPT	Age	CCI	−0.038 (−0.103–0.024)	0.112 (0.048–0.173)	0.150 (0.067–0.244)	True
GPT	Age	BMI	−0.054 (−0.117–0.004)	−0.150 (−0.212–−0.087)	−0.096 (−0.180–−0.009)	False
GPT	Age	Admission_BI	−0.186 (−0.246–−0.128)	−0.173 (−0.235–−0.112)	0.012 (−0.070–0.093)	False
GPT	Age	Days_to_Rehabilitation	0.008 (−0.054–0.065)	0.035 (−0.027–0.103)	0.027 (−0.054–0.123)	False
GPT	Age	Days_to_Surgery	−0.062 (−0.125–0.005)	0.034 (−0.028–0.092)	0.096 (0.011–0.183)	True
GPT	Age	Rehabilitation_Units	−0.038 (−0.096–0.020)	−0.047 (−0.107–0.014)	−0.008 (−0.091–0.073)	False
GPT	Age	Discharge_BI	−0.383 (−0.432–−0.328)	−0.221 (−0.278–−0.162)	0.162 (0.086–0.238)	False
GPT	Age	Length_of_Stay	0.009 (−0.049–0.067)	−0.005 (−0.066–0.058)	−0.014 (−0.106–0.073)	True
GPT	CCI	BMI	0.031 (−0.035–0.095)	−0.079 (−0.142–−0.015)	−0.109 (−0.199–−0.023)	True
GPT	CCI	Admission_BI	0.032 (−0.032–0.093)	−0.131 (−0.186–−0.073)	−0.163 (−0.242–−0.076)	True
GPT	CCI	Days_to_Rehabilitation	−0.043 (−0.102–0.019)	0.102 (0.040–0.160)	0.146 (0.062–0.230)	True
GPT	CCI	Days_to_Surgery	0.055 (−0.002–0.117)	0.171 (0.116–0.225)	0.116 (0.040–0.195)	False
GPT	CCI	Rehabilitation_Units	−0.020 (−0.081–0.041)	−0.077 (−0.133–−0.016)	−0.057 (−0.139–0.027)	False
GPT	CCI	Discharge_BI	−0.073 (−0.135–−0.007)	−0.196 (−0.257–−0.137)	−0.123 (−0.206–−0.038)	False
GPT	CCI	Length_of_Stay	−0.005 (−0.067–0.055)	0.058 (−0.002–0.117)	0.063 (−0.025–0.151)	True
GPT	BMI	Admission_BI	0.080 (0.018–0.140)	0.057 (−0.003–0.117)	−0.023 (−0.116–0.059)	False
GPT	BMI	Days_to_Rehabilitation	0.002 (−0.057–0.064)	−0.005 (−0.071–0.058)	−0.007 (−0.096–0.078)	True
GPT	BMI	Days_to_Surgery	−0.034 (−0.098–0.028)	−0.002 (−0.065–0.059)	0.033 (−0.058–0.126)	False
GPT	BMI	Rehabilitation_Units	0.068 (0.005–0.129)	0.014 (−0.048–0.072)	−0.054 (−0.141–0.031)	False
GPT	BMI	Discharge_BI	0.071 (0.006–0.131)	0.053 (−0.009–0.111)	−0.018 (−0.101–0.067)	False
GPT	BMI	Length_of_Stay	0.058 (−0.010–0.118)	−0.048 (−0.108–0.013)	−0.105 (−0.188–−0.014)	True
GPT	Admission_BI	Days_to_Rehabilitation	−0.052 (−0.110–0.013)	0.044 (−0.022–0.109)	0.095 (−0.002–0.184)	True
GPT	Admission_BI	Days_to_Surgery	0.005 (−0.059–0.065)	0.004 (−0.063–0.066)	−0.001 (−0.093–0.085)	False
GPT	Admission_BI	Rehabilitation_Units	0.078 (0.012–0.138)	0.035 (−0.026–0.097)	−0.043 (−0.126–0.045)	False
GPT	Admission_BI	Discharge_BI	0.357 (0.300–0.413)	0.257 (0.193–0.323)	−0.100 (−0.181–−0.013)	False
GPT	Admission_BI	Length_of_Stay	0.022 (−0.044–0.089)	−0.041 (−0.104–0.024)	−0.062 (−0.153–0.032)	True
GPT	Days_to_Rehabilitation	Days_to_Surgery	0.295 (0.234–0.353)	0.705 (0.668–0.740)	0.411 (0.346–0.487)	False
GPT	Days_to_Rehabilitation	Rehabilitation_Units	−0.095 (−0.150–−0.034)	−0.034 (−0.094–0.031)	0.060 (−0.020–0.140)	False
GPT	Days_to_Rehabilitation	Discharge_BI	−0.039 (−0.098–0.023)	−0.084 (−0.144–−0.023)	−0.045 (−0.131–0.036)	False
GPT	Days_to_Rehabilitation	Length_of_Stay	0.114 (0.053–0.175)	0.161 (0.102–0.222)	0.047 (−0.037–0.134)	False
GPT	Days_to_Surgery	Rehabilitation_Units	0.150 (0.092–0.212)	−0.003 (−0.065–0.061)	−0.153 (−0.243–−0.076)	True
GPT	Days_to_Surgery	Discharge_BI	−0.057 (−0.121–0.006)	−0.102 (−0.163–−0.040)	−0.045 (−0.132–0.045)	False
GPT	Days_to_Surgery	Length_of_Stay	0.249 (0.190–0.306)	0.169 (0.112–0.229)	−0.080 (−0.164–0.005)	False
GPT	Rehabilitation_Units	Discharge_BI	0.179 (0.118–0.241)	0.279 (0.220–0.336)	0.100 (0.017–0.184)	False
GPT	Rehabilitation_Units	Length_of_Stay	0.729 (0.692–0.765)	0.407 (0.358–0.457)	−0.322 (−0.385–−0.260)	False
GPT	Discharge_BI	Length_of_Stay	0.163 (0.102–0.222)	0.077 (0.016–0.138)	−0.086 (−0.170–0.001)	False
Claude	Age	CCI	−0.038 (−0.099–0.023)	−0.017 (−0.081–0.046)	0.021 (−0.071–0.111)	False
Claude	Age	BMI	−0.054 (−0.114–0.007)	0.030 (−0.031–0.092)	0.085 (−0.007–0.167)	True
Claude	Age	Admission_BI	−0.186 (−0.248–−0.124)	0.005 (−0.061–0.065)	0.190 (0.103–0.274)	True
Claude	Age	Days_to_Rehabilitation	0.008 (−0.055–0.072)	−0.002 (−0.062–0.058)	−0.010 (−0.095–0.078)	True
Claude	Age	Days_to_Surgery	−0.062 (−0.127–0.003)	−0.009 (−0.068–0.052)	0.052 (−0.032–0.150)	False
Claude	Age	Rehabilitation_Units	−0.038 (−0.098–0.021)	0.025 (−0.038–0.086)	0.063 (−0.027–0.150)	True

Claude	Age	Discharge_BI	−0.383 (−0.434–−0.327)	−0.104 (−0.167–−0.043)	0.279 (0.195–0.358)	False
Claude	Age	Length_of_Stay	0.009 (−0.052–0.066)	0.019 (−0.045–0.078)	0.010 (−0.077–0.095)	False
Claude	CCI	BMI	0.031 (−0.032–0.091)	0.013 (−0.053–0.075)	−0.018 (−0.111–0.077)	False
Claude	CCI	Admission_BI	0.032 (−0.027–0.094)	0.033 (−0.026–0.092)	0.001 (−0.085–0.084)	False
Claude	CCI	Days_to_Rehabilitation	−0.043 (−0.104–0.016)	0.008 (−0.055–0.071)	0.052 (−0.035–0.135)	True
Claude	CCI	Days_to_Surgery	0.055 (−0.004–0.117)	−0.024 (−0.089–0.037)	−0.080 (−0.167–−0.001)	True
Claude	CCI	Rehabilitation_Units	−0.020 (−0.077–0.041)	0.062 (0.002–0.126)	0.082 (−0.005–0.169)	True
Claude	CCI	Discharge_BI	−0.073 (−0.135–−0.016)	−0.018 (−0.075–0.042)	0.055 (−0.027–0.141)	False
Claude	CCI	Length_of_Stay	−0.005 (−0.067–0.055)	−0.010 (−0.070–0.055)	−0.005 (−0.088–0.084)	False
Claude	BMI	Admission_BI	0.080 (0.015–0.139)	0.008 (−0.054–0.077)	−0.072 (−0.155–0.018)	False
Claude	BMI	Days_to_Rehabilitation	0.002 (−0.057–0.060)	−0.015 (−0.076–0.048)	−0.017 (−0.103–0.070)	True
Claude	BMI	Days_to_Surgery	−0.034 (−0.098–0.026)	−0.046 (−0.110–0.016)	−0.012 (−0.100–0.073)	False
Claude	BMI	Rehabilitation_Units	0.068 (0.008–0.131)	−0.008 (−0.064–0.053)	−0.076 (−0.163–0.009)	True
Claude	BMI	Discharge_BI	0.071 (0.006–0.132)	0.021 (−0.042–0.088)	−0.050 (−0.142–0.042)	False
Claude	BMI	Length_of_Stay	0.058 (−0.004–0.123)	0.003 (−0.058–0.066)	−0.055 (−0.143–0.037)	False
Claude	Admission_BI	Days_to_Rehabilitation	−0.052 (−0.114–0.005)	−0.013 (−0.072–0.048)	0.039 (−0.044–0.125)	False
Claude	Admission_BI	Days_to_Surgery	0.005 (−0.059–0.065)	−0.007 (−0.070–0.057)	−0.012 (−0.094–0.077)	True
Claude	Admission_BI	Rehabilitation_Units	0.078 (0.016–0.142)	−0.027 (−0.088–0.036)	−0.105 (−0.195–−0.021)	True
Claude	Admission_BI	Discharge_BI	0.357 (0.301–0.410)	0.600 (0.554–0.641)	0.243 (0.170–0.308)	False
Claude	Admission_BI	Length_of_Stay	0.022 (−0.039–0.087)	−0.055 (−0.118–0.005)	−0.077 (−0.164–0.005)	True
Claude	Days_to_Rehabilitation	Days_to_Surgery	0.295 (0.228–0.355)	0.775 (0.739–0.811)	0.481 (0.410–0.555)	False
Claude	Days_to_Rehabilitation	Rehabilitation_Units	−0.095 (−0.152–−0.037)	−0.016 (−0.079–0.048)	0.079 (−0.005–0.165)	False
Claude	Days_to_Rehabilitation	Discharge_BI	−0.039 (−0.105–0.021)	−0.011 (−0.070–0.050)	0.028 (−0.056–0.120)	False
Claude	Days_to_Rehabilitation	Length_of_Stay	0.114 (0.049–0.171)	0.018 (−0.047–0.079)	−0.096 (−0.187–−0.003)	False
Claude	Days_to_Surgery	Rehabilitation_Units	0.150 (0.089–0.207)	−0.008 (−0.069–0.053)	−0.158 (−0.243–−0.071)	True
Claude	Days_to_Surgery	Discharge_BI	−0.057 (−0.114–0.005)	0.011 (−0.051–0.072)	0.068 (−0.020–0.154)	True
Claude	Days_to_Surgery	Length_of_Stay	0.249 (0.191–0.304)	0.013 (−0.050–0.070)	−0.235 (−0.322–−0.150)	False
Claude	Rehabilitation_Units	Discharge_BI	0.179 (0.119–0.237)	0.443 (0.386–0.496)	0.265 (0.182–0.345)	False
Claude	Rehabilitation_Units	Length_of_Stay	0.729 (0.693–0.763)	0.016 (−0.048–0.075)	−0.713 (−0.788–−0.646)	False
Claude	Discharge_BI	Length_of_Stay	0.163 (0.102–0.225)	−0.006 (−0.070–0.053)	−0.169 (−0.253–−0.085)	True
Gemini	Age	CCI	−0.038 (−0.099–0.019)	0.166 (0.103–0.229)	0.204 (0.117–0.290)	True
Gemini	Age	BMI	−0.054 (−0.115–0.011)	−0.087 (−0.144–−0.023)	−0.033 (−0.122–0.059)	False
Gemini	Age	Admission_BI	−0.186 (−0.248–−0.122)	−0.190 (−0.252–−0.131)	−0.004 (−0.090–0.080)	False
Gemini	Age	Days_to_Rehabilitation	0.008 (−0.057–0.069)	0.012 (−0.050–0.071)	0.005 (−0.083–0.088)	False
Gemini	Age	Days_to_Surgery	−0.062 (−0.129–0.001)	0.017 (−0.047–0.083)	0.078 (−0.009–0.164)	True
Gemini	Age	Rehabilitation_Units	−0.038 (−0.100–0.022)	−0.050 (−0.114–0.010)	−0.012 (−0.092–0.069)	False
Gemini	Age	Discharge_BI	−0.383 (−0.432–−0.329)	−0.248 (−0.305–−0.192)	0.135 (0.060–0.211)	False
Gemini	Age	Length_of_Stay	0.009 (−0.052–0.071)	0.140 (0.077–0.202)	0.131 (0.045–0.215)	False
Gemini	CCI	BMI	0.031 (−0.036–0.089)	−0.117 (−0.175–−0.053)	−0.147 (−0.225–−0.058)	True
Gemini	CCI	Admission_BI	0.032 (−0.027–0.094)	−0.149 (−0.205–−0.092)	−0.181 (−0.263–−0.094)	True
Gemini	CCI	Days_to_Rehabilitation	−0.043 (−0.105–0.018)	0.091 (0.029–0.148)	0.134 (0.051–0.218)	True
Gemini	CCI	Days_to_Surgery	0.055 (−0.002–0.115)	0.229 (0.170–0.290)	0.174 (0.088–0.256)	False
Gemini	CCI	Rehabilitation_Units	−0.020 (−0.083–0.041)	0.085 (0.021–0.143)	0.105 (0.023–0.193)	True
Gemini	CCI	Discharge_BI	−0.073 (−0.130–−0.014)	−0.174 (−0.234–−0.114)	−0.101 (−0.190–−0.014)	False

Gemini	CCI	Length_of_Stay	−0.005 (−0.067–0.057)	0.172 (0.107–0.226)	0.176 (0.091–0.260)	True
Gemini	BMI	Admission_BI	0.080 (0.022–0.145)	0.074 (0.009–0.140)	−0.006 (−0.096–0.083)	False
Gemini	BMI	Days_to_Rehabilitation	0.002 (−0.060–0.065)	−0.057 (−0.115–0.003)	−0.059 (−0.141–0.030)	True
Gemini	BMI	Days_to_Surgery	−0.034 (−0.096–0.027)	−0.053 (−0.117–0.012)	−0.019 (−0.110–0.081)	False
Gemini	BMI	Rehabilitation_Units	0.068 (0.005–0.128)	−0.007 (−0.068–0.056)	−0.075 (−0.157–0.021)	True
Gemini	BMI	Discharge_BI	0.071 (0.009–0.135)	0.100 (0.040–0.165)	0.030 (−0.060–0.110)	False
Gemini	BMI	Length_of_Stay	0.058 (−0.003–0.118)	−0.112 (−0.170–−0.048)	−0.170 (−0.258–−0.075)	True
Gemini	Admission_BI	Days_to_Rehabilitation	−0.052 (−0.113–0.009)	−0.059 (−0.116–0.006)	−0.007 (−0.091–0.080)	False
Gemini	Admission_BI	Days_to_Surgery	0.005 (−0.053–0.066)	−0.039 (−0.099–0.025)	−0.044 (−0.132–0.042)	True
Gemini	Admission_BI	Rehabilitation_Units	0.078 (0.014–0.142)	−0.065 (−0.125–−0.005)	−0.142 (−0.230–−0.053)	True
Gemini	Admission_BI	Discharge_BI	0.357 (0.297–0.411)	0.581 (0.543–0.622)	0.224 (0.153–0.298)	False
Gemini	Admission_BI	Length_of_Stay	0.022 (−0.039–0.083)	−0.206 (−0.263–−0.145)	−0.228 (−0.311–−0.140)	True
Gemini	Days_to_Rehabilitation	Days_to_Surgery	0.295 (0.229–0.357)	0.422 (0.364–0.475)	0.128 (0.044–0.209)	False
Gemini	Days_to_Rehabilitation	Rehabilitation_Units	−0.095 (−0.156–−0.035)	0.167 (0.110–0.226)	0.262 (0.176–0.343)	True
Gemini	Days_to_Rehabilitation	Discharge_BI	−0.039 (−0.102–0.022)	−0.114 (−0.173–−0.051)	−0.075 (−0.163–0.014)	False
Gemini	Days_to_Rehabilitation	Length_of_Stay	0.114 (0.049–0.176)	0.253 (0.194–0.309)	0.139 (0.054–0.220)	False
Gemini	Days_to_Surgery	Rehabilitation_Units	0.150 (0.091–0.210)	0.146 (0.088–0.207)	−0.004 (−0.087–0.081)	False
Gemini	Days_to_Surgery	Discharge_BI	−0.057 (−0.120–0.007)	−0.131 (−0.193–−0.066)	−0.074 (−0.167–0.019)	False
Gemini	Days_to_Surgery	Length_of_Stay	0.249 (0.195–0.307)	0.215 (0.151–0.272)	−0.034 (−0.114–0.047)	False
Gemini	Rehabilitation_Units	Discharge_BI	0.179 (0.119–0.239)	0.094 (0.029–0.154)	−0.085 (−0.173–−0.000)	False
Gemini	Rehabilitation_Units	Length_of_Stay	0.729 (0.691–0.763)	0.837 (0.814–0.856)	0.108 (0.067–0.150)	False
Gemini	Discharge_BI	Length_of_Stay	0.163 (0.101–0.223)	−0.089 (−0.150–−0.030)	−0.252 (−0.338–−0.172)	True

Note: Each correlation was calculated using 1,000 records. Signed difference was defined as the synthetic-data Spearman correlation minus the real-data correlation. Absolute differences were used for the model-level summary measures reported in the main text.

Table S4. Operational plausibility checks with nonzero findings

Dataset	Rule	Count	Percent
GPT	Trochanteric fracture with hemiarthroplasty	44	4.4%
Claude	Rehabilitation starts on or after discharge	1	0.1%
Claude	Rehabilitation units exceed the limit of 9 units per available day	24	2.4%
Claude	Trochanteric fracture with hemiarthroplasty	4	0.4%

Table S5A. Exploratory discharge BI standardized coefficients

Dataset	Predictor	Standardized beta	95% CI
Real	Age	−0.346	−0.399–−0.285
Real	Male	−0.019	−0.074–0.034
Real	Trochanteric_fracture	−0.065	−0.153–0.015
Real	Hemiarthroplasty	0.010	−0.074–0.093
Real	CCI	−0.090	−0.142–−0.039
Real	BMI	0.036	−0.023–0.089
Real	Admission_BI	0.285	0.234–0.339
Real	Days_to_Rehabilitation	−0.015	−0.099–0.037
Real	Days_to_Surgery	−0.120	−0.177–−0.064
Real	Rehabilitation_Units	0.007	−0.100–0.117
Real	Length_of_Stay	0.165	0.080–0.263
GPT	Age	−0.156	−0.208–−0.099
GPT	Male	−0.004	−0.058–0.052
GPT	Trochanteric_fracture	0.021	−0.051–0.091
GPT	Hemiarthroplasty	0.026	−0.044–0.090

GPT	CCI	−0.110	−0.170–−0.061
GPT	BMI	0.013	−0.041–0.066
GPT	Admission_BI	0.292	0.230–0.349
GPT	Days_to_Rehabilitation	−0.060	−0.131–0.018
GPT	Days_to_Surgery	−0.036	−0.115–0.031
GPT	Rehabilitation_Units	0.234	0.176–0.293
GPT	Length_of_Stay	0.013	−0.058–0.081
Claude	Age	−0.116	−0.152–−0.081
Claude	Male	−0.000	−0.040–0.035
Claude	Trochanteric_fracture	0.024	−0.037–0.086
Claude	Hemiarthroplasty	0.010	−0.054–0.075
Claude	CCI	−0.056	−0.089–−0.021
Claude	BMI	0.028	−0.013–0.065
Claude	Admission_BI	0.647	0.610–0.688
Claude	Days_to_Rehabilitation	0.017	−0.068–0.103
Claude	Days_to_Surgery	−0.009	−0.092–0.076
Claude	Rehabilitation_Units	0.487	0.411–0.600
Claude	Length_of_Stay	0.004	−0.029–0.040
Gemini	Age	−0.119	−0.171–−0.065
Gemini	Male	0.021	−0.026–0.073
Gemini	Trochanteric_fracture	0.034	−0.045–0.107
Gemini	Hemiarthroplasty	−0.045	−0.124–0.027
Gemini	CCI	−0.030	−0.082–0.024
Gemini	BMI	0.064	0.013–0.116
Gemini	Admission_BI	0.523	0.488–0.559
Gemini	Days_to_Rehabilitation	−0.049	−0.138–0.010
Gemini	Days_to_Surgery	−0.071	−0.147–−0.016
Gemini	Rehabilitation_Units	0.139	0.048–0.241
Gemini	Length_of_Stay	−0.060	−0.150–0.022

Table S5B. Exploratory discharge BI predictive performance

Training data	Evaluation data	Method	RMSE	R-squared
Real	Real	5-fold CV	28.84	0.262
GPT	GPT	5-fold CV	22.44	0.209
Claude	Claude	5-fold CV	20.87	0.634
Gemini	Gemini	5-fold CV	27.12	0.348
GPT	Real	Train synthetic / test real	30.64	0.167
Claude	Real	Train synthetic / test real	34.89	−0.080
Gemini	Real	Train synthetic / test real	31.03	0.146

Table S6. Distributional diagnostics

Dataset	Variable	Zero %	Minimum n	Q25	Median	Q75	Maximum n
Real	Age	0.0	3	81.00	87.00	91.00	15
Real	CCI	43.7	437	0.00	1.00	1.00	1
Real	BMI	0.0	1	18.20	20.10	22.20	1
Real	Admission_BI	36.4	364	0.00	5.00	15.00	54
Real	Days_to_Rehabilitation	6.7	67	1.00	2.00	3.00	1
Real	Days_to_Surgery	14.9	149	1.00	2.00	4.00	1
Real	Rehabilitation_Units	0.0	2	13.00	22.00	40.00	1
Real	Discharge_BI	8.7	87	15.00	45.00	75.00	115
Real	Length_of_Stay	0.0	2	18.00	24.00	36.00	1
GPT	Age	0.0	5	81.00	86.00	91.00	31
GPT	CCI	56.2	562	0.00	0.00	1.00	1
GPT	BMI	0.0	3	18.70	20.50	22.30	1
GPT	Admission_BI	75.5	755	0.00	0.00	0.00	1
GPT	Days_to_Rehabilitation	23.0	230	1.00	2.00	4.00	1
GPT	Days_to_Surgery	9.6	96	1.00	2.00	3.00	1
GPT	Rehabilitation_Units	0.0	3	18.00	34.00	62.00	1
GPT	Discharge_BI	4.0	40	30.00	45.00	65.00	33
GPT	Length_of_Stay	0.0	1	28.00	36.00	48.00	1
Claude	Age	0.0	4	80.00	86.00	91.00	30
Claude	CCI	46.7	467	0.00	1.00	1.00	1
Claude	BMI	0.0	7	18.30	20.20	22.30	1
Claude	Admission_BI	58.7	587	0.00	0.00	20.00	25
Claude	Days_to_Rehabilitation	4.1	41	1.00	2.00	3.00	1
Claude	Days_to_Surgery	17.2	172	1.00	2.00	4.00	1
Claude	Rehabilitation_Units	0.0	3	12.00	22.50	43.00	3
Claude	Discharge_BI	3.8	38	15.00	40.00	80.00	191
Claude	Length_of_Stay	0.0	3	17.00	26.00	40.00	1
Gemini	Age	0.0	1	81.00	87.00	91.00	1
Gemini	CCI	42.8	428	0.00	1.00	1.00	1

Gemini	BMI	0.0	1	18.40	20.40	22.30	1
Gemini	Admission_BI	64.7	647	0.00	0.00	26.25	1
Gemini	Days_to_Rehabilitation	5.9	59	1.00	2.00	3.00	1
Gemini	Days_to_Surgery	18.8	188	1.00	2.00	4.00	1
Gemini	Rehabilitation_Units	0.0	1	11.75	21.00	42.00	1
Gemini	Discharge_BI	14.3	143	15.00	45.00	75.00	84
Gemini	Length_of_Stay	0.0	18	16.00	26.00	42.00	1

Figure S1. Patient-selection flow

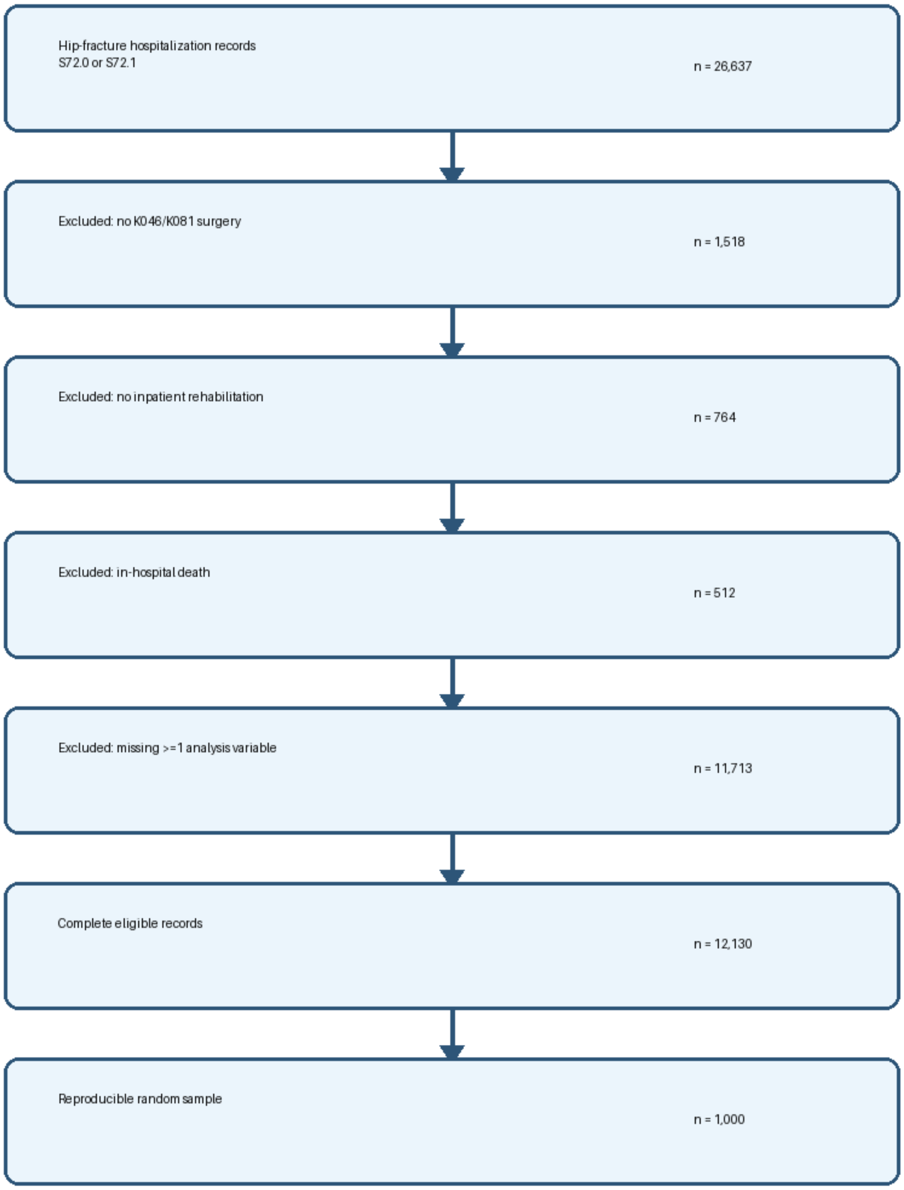

Figure S2. Real DPC data correlation heatmap

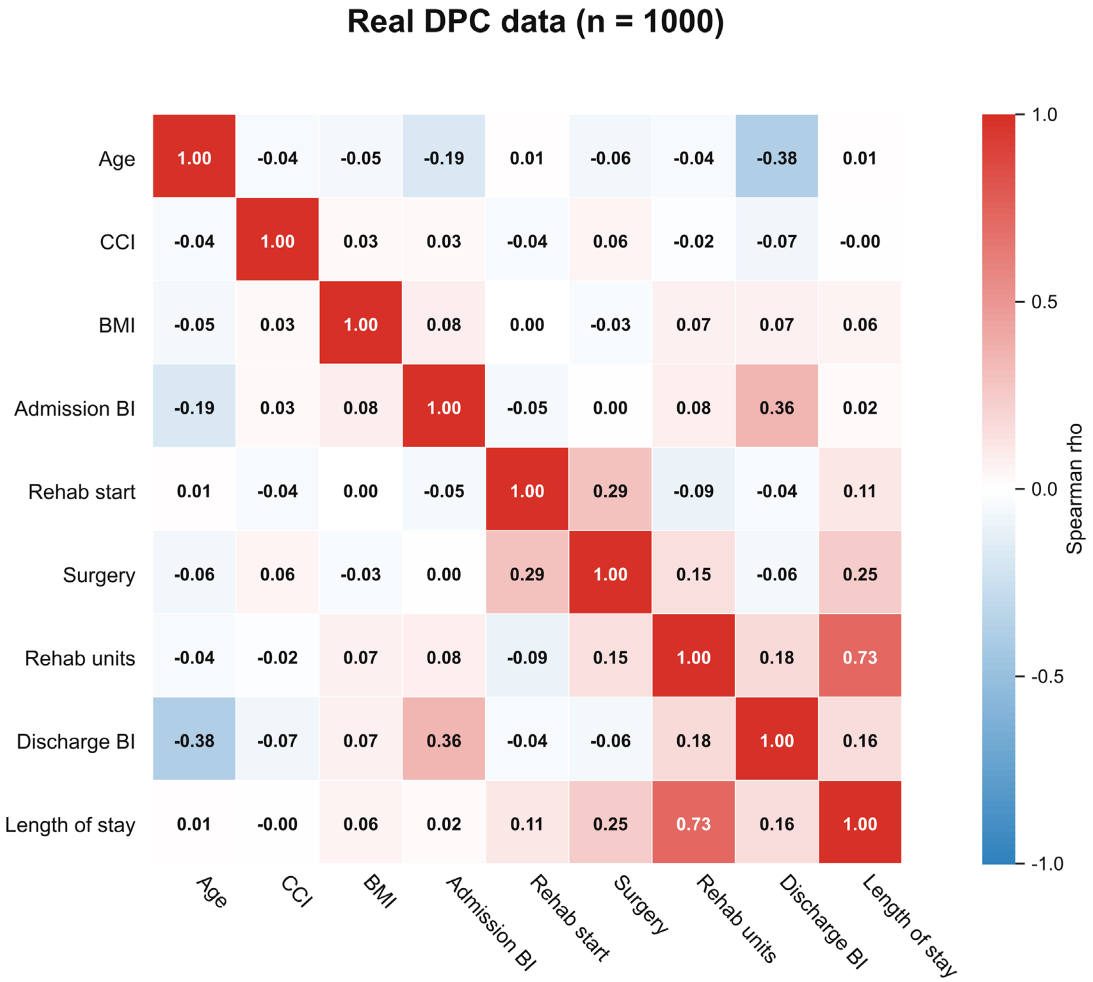

Figure S3. GPT correlation heatmap

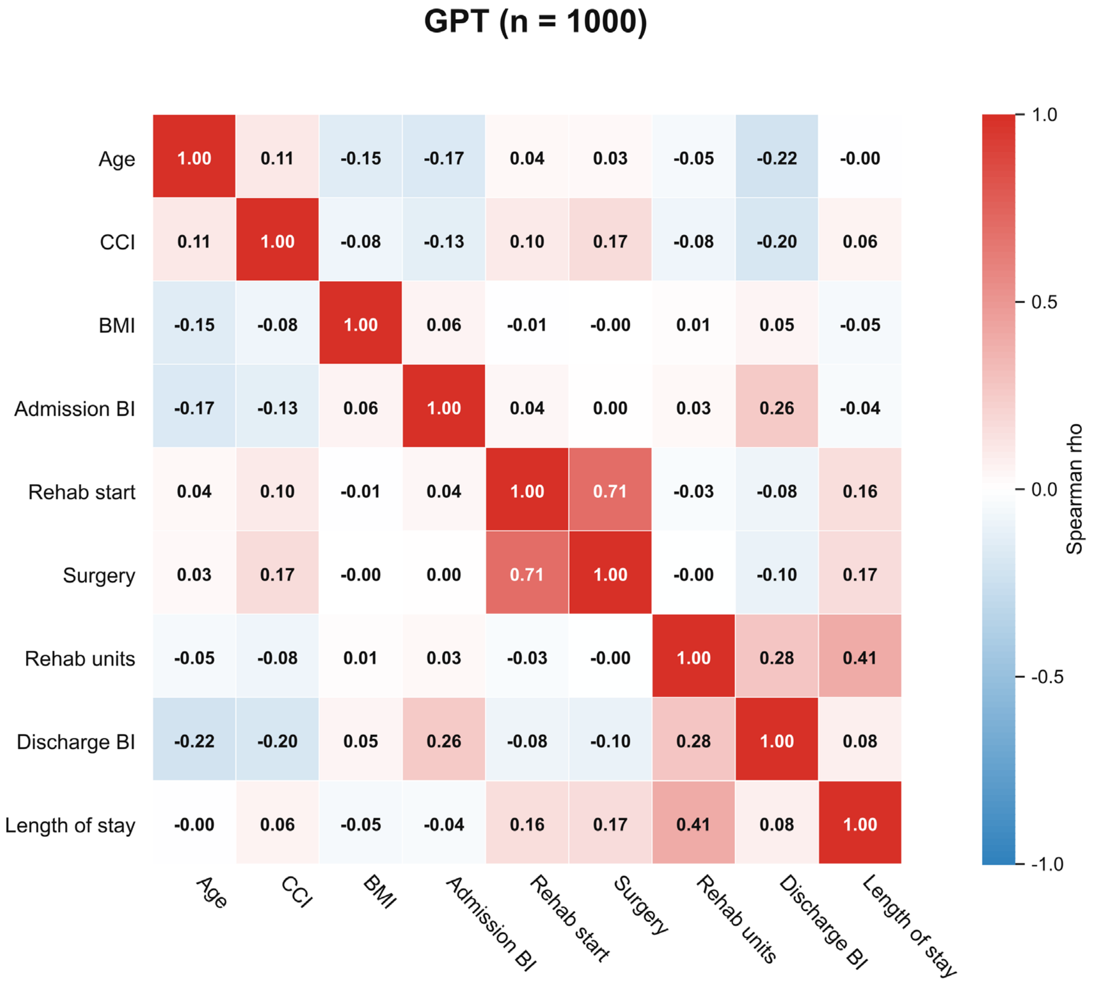

Figure S4. Claude correlation heatmap

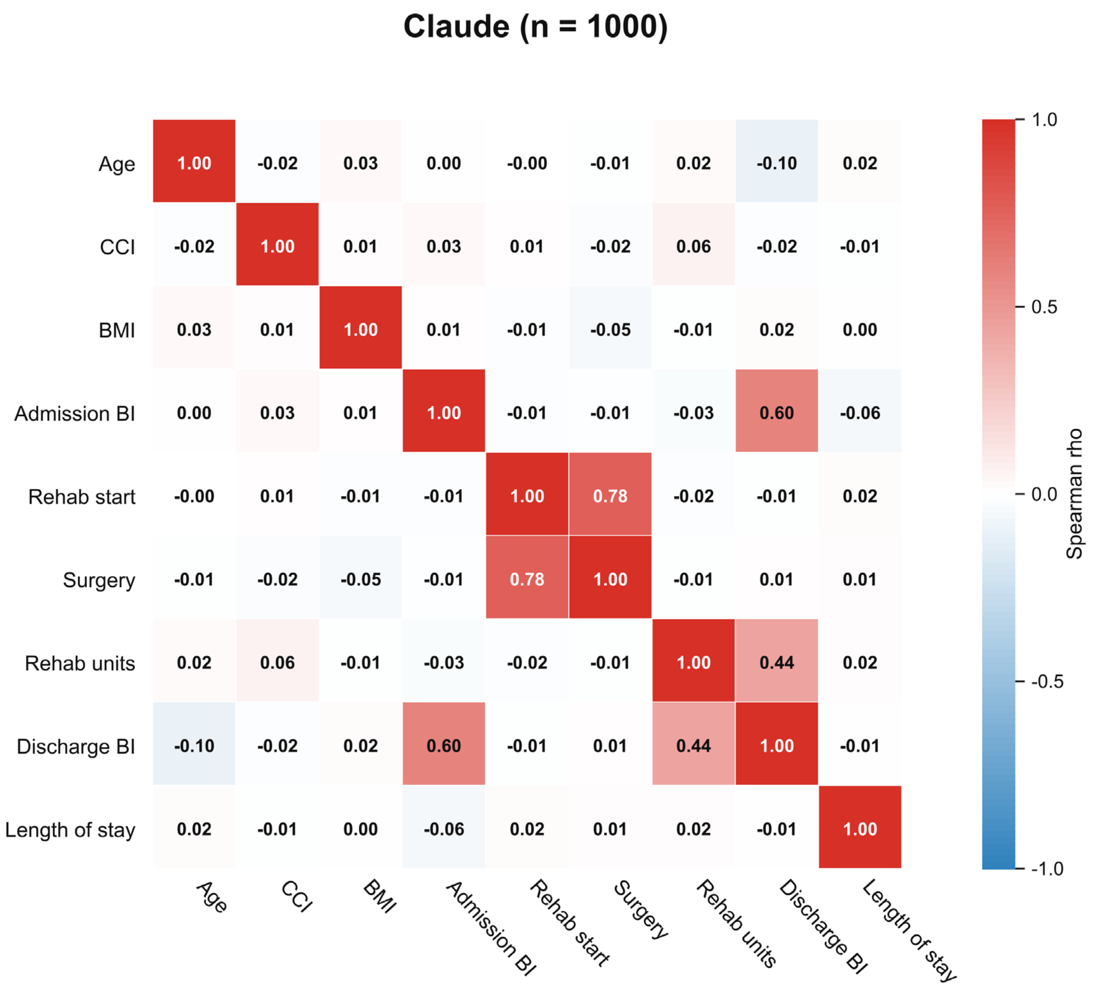

Figure S5. Gemini correlation heatmap

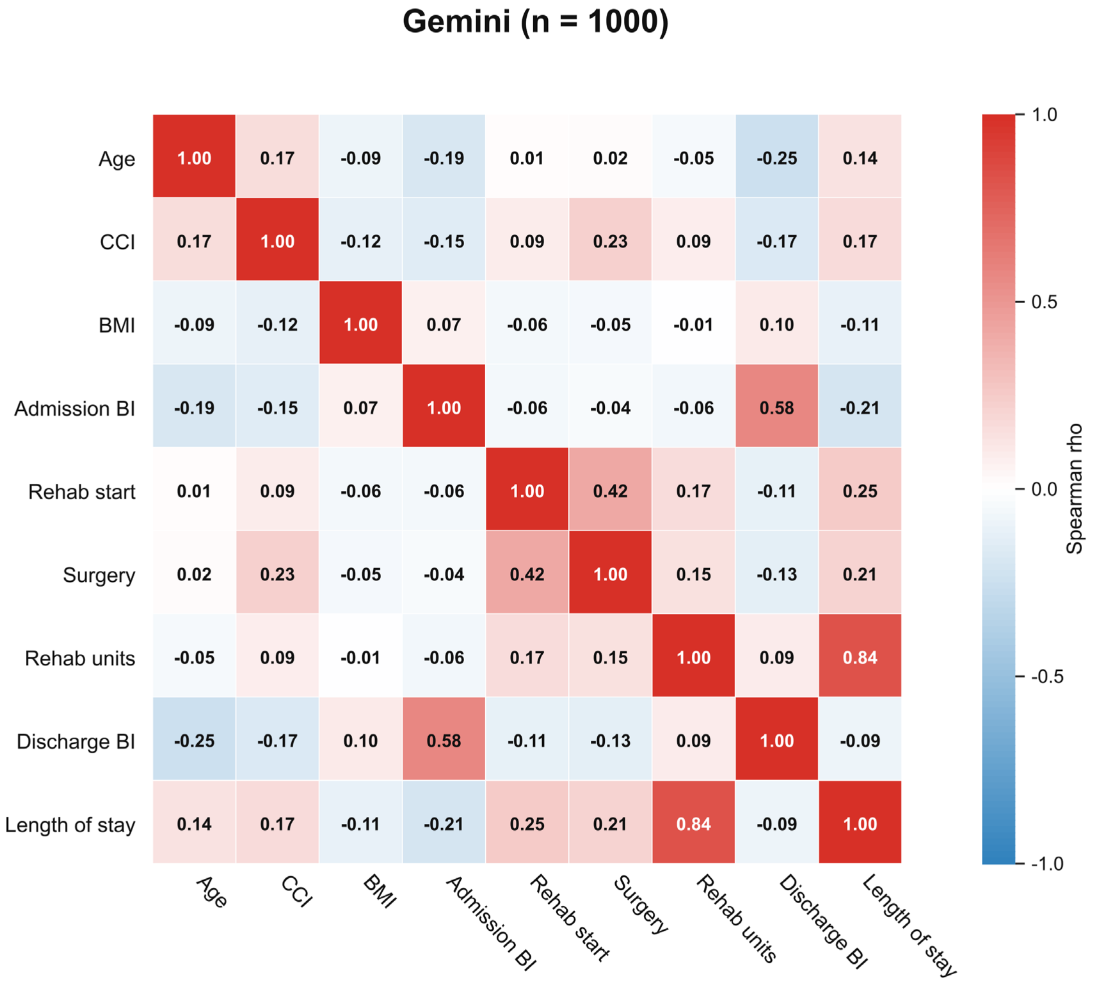

Figure S6. GPT minus real-data correlation-difference heatmap

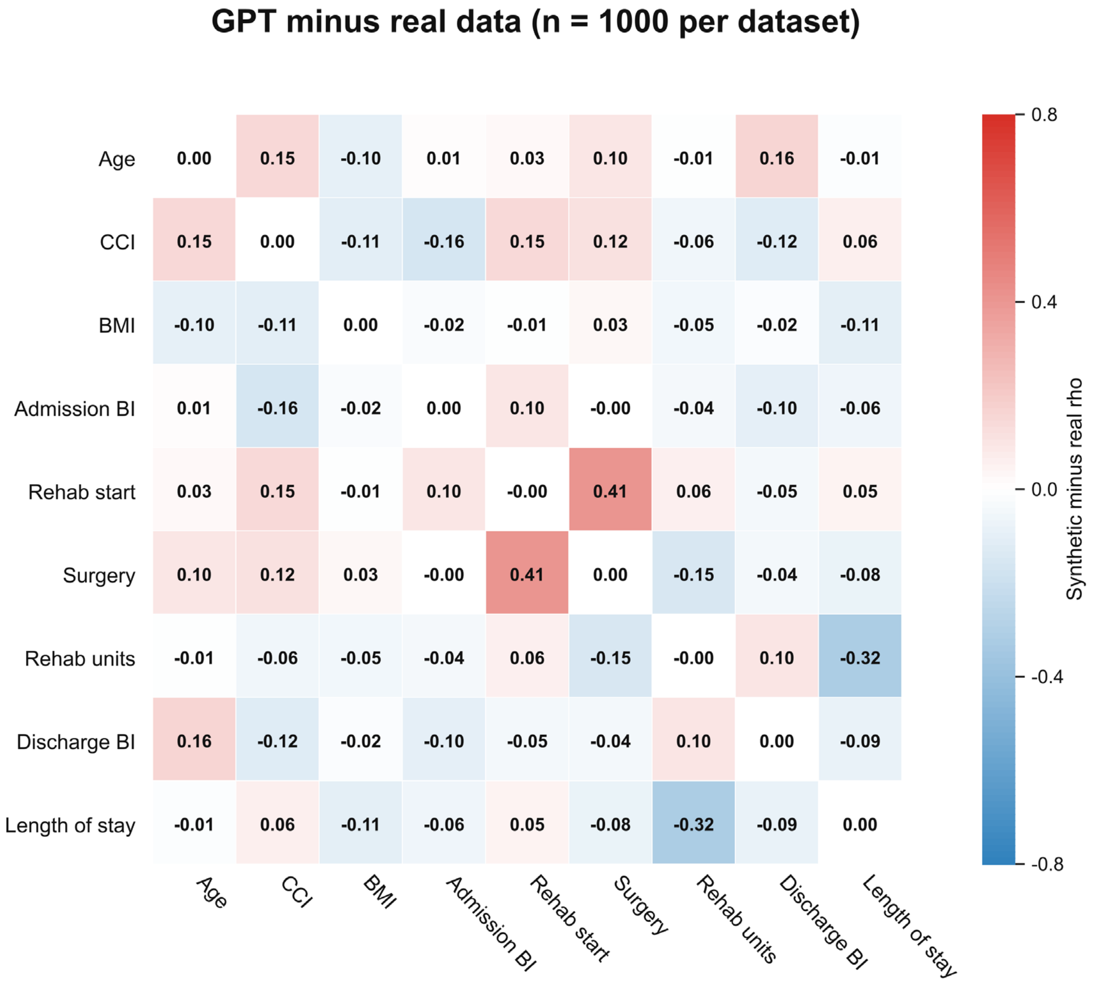

Figure S7. Claude minus real-data correlation-difference heatmap

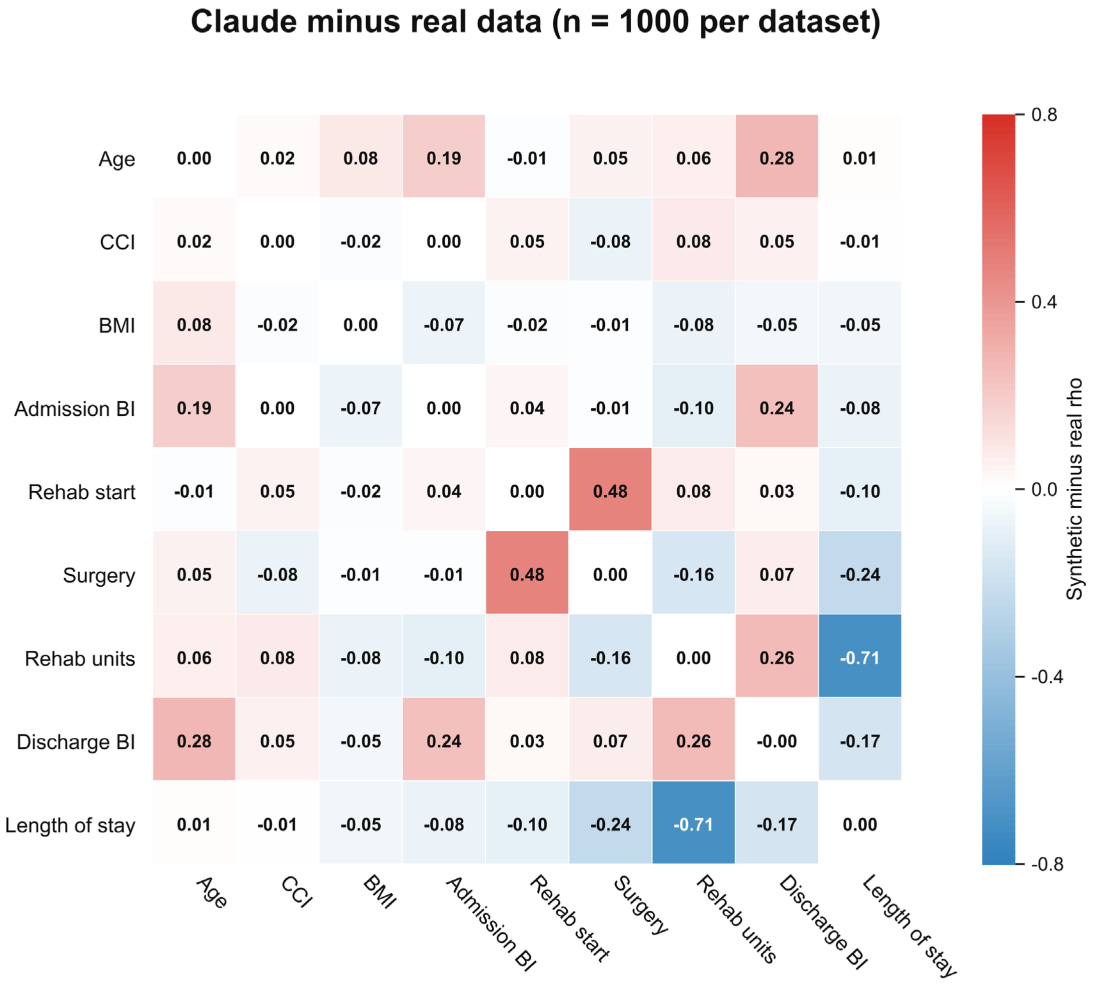

Figure S8. Gemini minus real-data correlation-difference heatmap

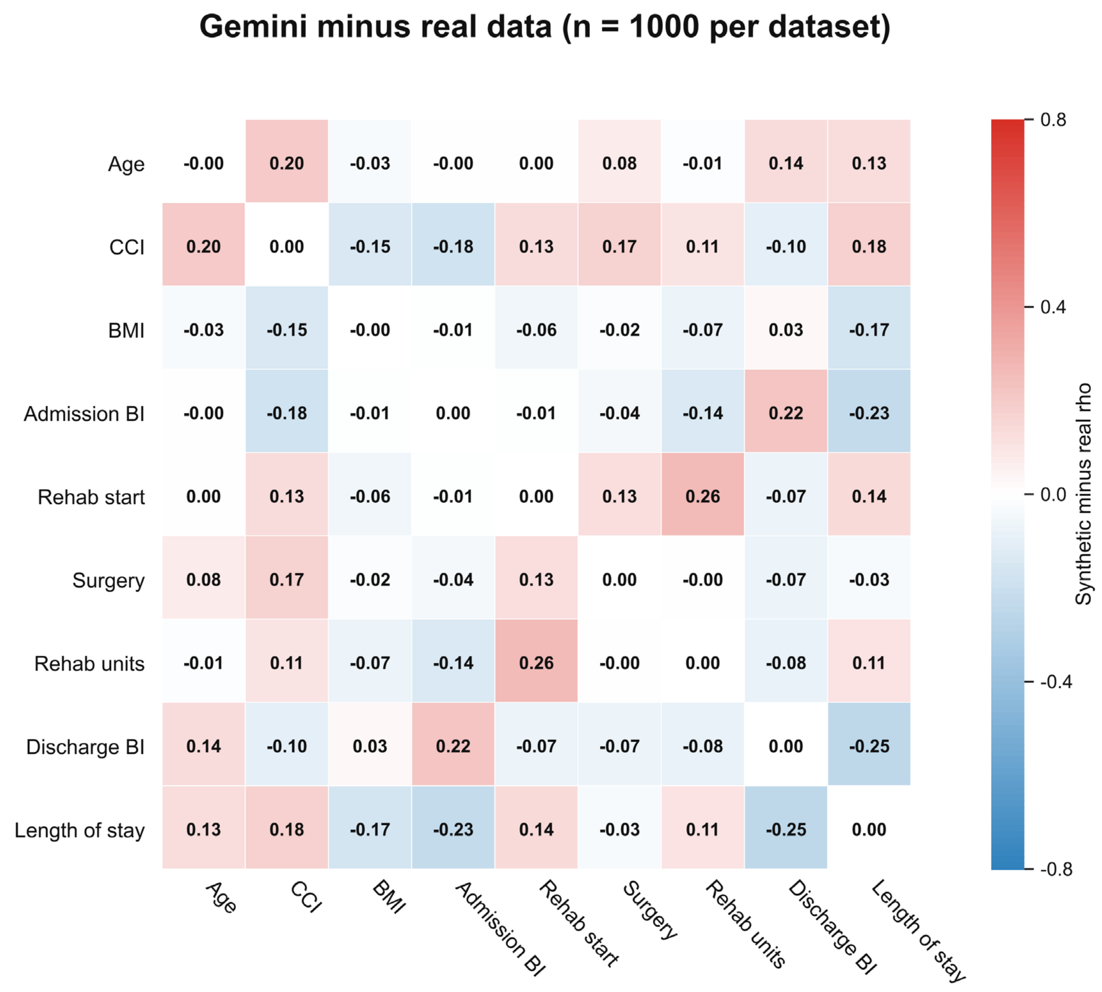

Supplement: Supplementary file 1 [file jcm-15-08700-s001.zip › Supplementary_Analyses.pdf]
