# Supplementary material for: Exploratory Evaluation of the Statistical Fidelity of Large Language Model-Generated Synthetic Rehabilitation Data for Patients with Hip Fracture
Source: J Clin Med. 2026 Nov 7;15(21):8700. doi: 10.3390/jcm15218700 (PMC13764035; doi:10.3390/jcm15218700)
Supplement: Supplementary file 1 [file jcm-15-08700-s001.zip › Text_S1.pdf]

Text S1. Prompt used for synthetic data generation.

Please generate a synthetic patient dataset representing patients with hip fracture in Japan.
The target population consists of patients who were hospitalized for hip fracture, underwent surgical treatment, and received inpatient rehabilitation.
The information provided below consists solely of variable names and coding labels and aggregate-level descriptive statistics derived from actual Japanese Diagnosis Procedure Combination (DPC) data; no individual-level patient data are included.
Based on this information, generate synthetic data for 1,000 patients that reflect the statistical characteristics of the actual patient population. In addition, ensure that the relationships among variables within individual patients are clinically plausible.
【Fracture Type】
- S72.0 (femoral neck fracture): 52.1%
- S72.1 (trochanteric fracture): 47.9%
【Surgical Procedure】
- K046 (open reduction and internal fixation for fracture): 60.3%
- K081 (hemiarthroplasty): 39.7%
【Sex】
- Female: 78.4%
- Male: 21.6%
【Age】
- Mean: 85.63 years
- Standard deviation: 7.63 years
- Range: 65–100 years
【Charlson Comorbidity Index】
- Mean: 0.93
- Standard deviation: 1.12
- Range: 0–9
【BMI at admission】
- Mean: 20.33 kg/m^2
- Standard deviation: 2.90 kg/m^2
- Range: 13.4–32.3 kg/m^2
【Barthel Index at Admission (Admission BI)】
- Mean: 15.88
- Standard deviation: 26.60
- Range: 0–100
【Days from Admission to Rehabilitation Initiation】
- Mean: 2.60 days
- Standard deviation: 2.37 days
- Range: 0–44 days
【Days from Admission to Surgery】
- Mean: 2.93 days
- Standard deviation: 3.29 days
- Range: 0–49 days
【Total Rehabilitation Units During Hospitalization】
- Mean: 35.61 units

- Standard deviation: 44.04 units
- Range: 1–416 units

【Barthel Index at Discharge (Discharge BI)】
- Mean: 46.46
- Standard deviation: 33.60
- Range: 0–100

【Length of Stay】
- Mean: 31.84 days
- Standard deviation: 22.43 days
- Range: 5–169 days

Based on the information above, generate one synthetic dataset containing 1,000 patients.
Each row should represent one patient. Output the dataset in CSV format using the following 12 columns:
Fracture_Type, Surgical_Procedure, Sex, Age, CCI, BMI, Admission_BI, Days_to_Rehabilitation, Days_to_Surgery, Rehabilitation_Units, Discharge_BI, Length_of_Stay
Output only the CSV dataset, without any explanations, summaries, or comments.
